# A novel two-component system contributing the catabolism of c-di-GMP influences virulence in *Aeromonas veronii*

**DOI:** 10.3389/fmicb.2025.1527317

**Published:** 2025-01-30

**Authors:** Chaolun Liu, Jia Shao, Xiang Ma, Yanqiong Tang, Juanjuan Li, Hong Li, Xue Chi, Zhu Liu

**Affiliations:** School of Life and Health Sciences, Hainan University, Haikou, China

**Keywords:** *Aeromonas veronii*, two-component system, c-di-GMP, phosphodiesterase, motility, ArgR, virulence

## Abstract

**Introduction:**

Response regulators from diverse two-component systems often function as diguanylate cyclases or phosphodiesterases, thereby enabling precise regulation of intracellular c-di-GMP levels to control bacterial virulence and motility. However, the regulatory mechanisms of c-di-GMP require further elucidation.

**Methods:**

This study confirmed that ArrS and ArrR form a two-component system via structural analysis, two-hybrid, and phosphodiesterase activity detection. To evaluate the impact of ArrS/ArrR on intracellular c-di-GMP levels, biofilm detection, motility detection, fluorescence reporter plasmids, and LC-MS/MS analysis were employed. One-hybrid, EMSA, and RT-qPCR were used to demonstrate the function of ArgR on arrSR promoter. The roles of ArrS/ArrR in *Aeromonas veronii* were investigated using RT-qPCR, murine model, and proteomics.

**Results:**

ArrS and ArrR constituted a two-component system in *Aeromonas veronii* and were transcriptionally repressed by ArgR. ArrR exhibited phosphodiesterase activity, which is inhibited through phosphorylation mediated by ArrS. In *Aeromonas veronii*, ArrS/ArrR significantly altered the intracellular c-di-GMP levels. In a murine model, Δ*arrS* exhibited increased pathogenicity, leading to elevated TNF-α and IFN-γ levels in serum, and severer toxicity to spleen and kidney. These effects might be elucidated by the upregulated inflammation-associated proteins in Δ*arrS*. Moreover, the exonuclease RecB was also up-regulated in Δ*arrS*.

**Discussion:**

We elucidated the regulatory mechanism of ArrS/ArrR on intracellular c-di-GMP levels and its impact on the virulence in *Aeromonas veronii*, and discussed the intricate relationship between c-di-GMP metabolism and arginine metabolism.

## Introduction

*Aeromonas veronii* is a facultative anaerobic, Gram-negative bacterium that possesses both a unipolar flagellar system and lateral flagella. It is widely distributed in diverse aquatic environments and exhibits robust survival and motility capabilities. Among clinical *Aeromonas* species, *A. veronii* stands out as one of the most prevalent, accounting for approximately one-quarter of all cases (Figueras and Beaz-Hidalgo, 2015). This bacterium possesses the capability to secrete exotoxins including hemolysin, cytotoxic enterotoxin, serine protease, and collagenase (Prediger et al., 2020). Human infections primarily occur through avenues such as water pollution, food contamination, and wound infections. Infections of *A. veronii* manifest with mild symptoms such as diarrhea and wound swelling; however, severe cases can progress to gastroenteritis, sepsis, and organ failure, posing a threat to public health (Fernandez-Bravo and Figueras, 2020). Regrettably, there is a lack of comprehensive studies on the pathogenesis of *A. veronii*, significantly impeding our capacity to devise effective preventive and therapeutic strategies.

In the majority of pathogenic bacteria, the intracellular concentration of the second messenger c-di-GMP serves as a determinant of their virulence status (Valentini and Filloux, 2019), orchestrating crucial virulence activities such as host cell adherence, secretion of virulence factors, cytotoxicity, invasion, evasion, and resistance to oxidative stress (Jenal et al., 2017; Hall and Lee, 2018). Furthermore, c-di-GMP also plays a crucial regulatory role in various cellular processes, such as motility, biofilm formation, development and morphogenesis (Jenal et al., 2017). Its synthesis and degradation are modulated by diguanylate cyclase (DGC) and phosphodiesterase (PDE). Given the critical importance of c-di-GMP, bacteria usually possess a diverse array of different DGCs and PDEs (Povolotsky and Hengge, 2012; Mata et al., 2018; Homma and Kojima, 2022). Additionally, signal input domains located in their N-terminals including the Per-ARNT-Sim (PAS) domain, the GAF domain, the HAMP domain, and the phosphoacceptor receiver (REC) domain (Schirmer, 2016), facilitate a swift and accurate modulation of intracellular c-di-GMP levels in response to various environmental and cellular signals. This intricate interplay forms a complex c-di-GMP regulatory network, contributing to the precise regulation of bacterial physiological responses (Galperin et al., 2018; Randall et al., 2022). Two-component system (TCS) constitutes a crucial element in the aforementioned c-di-GMP regulatory network, which generally comprises a sensor kinase (SK) and a response regulator (RR). The classical SK usually comprises sensor domains, a histidine kinase (HK) domain and a histidine phosphotransfer (HPT) domain. These structures enable SK to perceive environmental signals and undergo autophosphorylation, which subsequently results in phosphotransfer to the RR. Typically, the RR is composed of an N-terminal regulatory domain and a C-terminal effector domain, and its biological function is dictated by the latter. For TCSs that associated with c-di-GMP modulations, certain RRs featuring GGDEF domains as their effector domain demonstrate DGC activity, including WspR, PleD, CfcR, CdgS (Galperin, 2010; Tagua et al., 2022; Sun et al., 2023); others possessing HD-GYP or EAL domains demonstrate PDE activity, including RocR, VieA, RavR, PvrR, and RpfG (Tamayo et al., 2005; Rao et al., 2009; Galperin, 2010; Cheng et al., 2019). Additionally, specific RRs, like Lpl0329 and Lpg0277, possess both types of the aforementioned domains and exhibit dual functionalities (Levet-Paulo et al., 2011; Hughes et al., 2019). Consequently, these TCSs constituted critical regulatory pathways for the c-di-GMP metabolism in bacteria (Junkermeier and Hengge, 2023). They actively engage in the synthesis or degradation of intracellular c-di-GMP, and serve as precise instruments that effectively translate various signals from the environments or cells into intracellular c-di-GMP concentrations, thereby eliciting specific cellular responses and enabling bacteria to sensitively adapt to changes in their surroundings. Unfortunately, despite structural analyses suggesting that many TCSs in *Aeromonas* are associated with c-di-GMP metabolism based on their effector domains (GGDEF, HD-GYP, and EAL), their specific functions and regulatory mechanisms have rarely been experimentally validated. In *A. veronii*, genome sequencing revealed the presence of 36 proteins containing the GGDEF domain, 16 proteins with the EAL domain, and 11 proteins with the HD-GYP domain. However, only the functions of UZE59342.1 (CsrD) and UZE59093.1 (AHA0701h) had been experimentally verified (Kozlova et al., 2012; Gladyshchuk et al., 2024).

In our preceding investigation, we identified an uncharacterized TCS in *A. veronii* (Liu et al., 2015), where its SK, denoted as ArrS and encoded by *ONR73_07150*, exhibited structural similarities with BvgS of *Bordetella pertussis*; and its RR, denoted as ArrR and encoded by *ONR73_07145*, featured an EAL domain at C-terminus. In this study, we confirmed the formation of a TCS where ArrS-mediated phosphorylation hindered the PDE activity of ArrR. Additionally, we presented cogent evidences supporting the involvement of ArrS/ArrR in intracellular c-di-GMP catabolism, showcasing its influence on biofilm formation, motility, and virulence in *A. veronii*. Moreover, we discovered an ARG-BOX motif situated within the −10 and −35 regions of the promoter of *arrS*, and identified ArgR as a transcriptional repressor for the *arrSR* operon. In conclusion, our findings identified a novel TCS associated with c-di-GMP metabolism and virulence, offering theoretical foundations for potential interventions against *A. veronii* infections.

## Methods

### Bacterial strains and culture conditions

Strains and plasmids employed in this study were detailed in Supplementary Table S1. The *E. coli* WM3064 strain, an auxotrophic strain that relies on diaminopimelic acid (DAP), was utilized as the donor strain for transferring plasmids into *A. veronii* through bacterial conjugation. The Δ*arrS* strain denoted an *A. veronii* mutant harboring a deletion mutation (from 271 to 668 bp) in *arrS* gene, whereas the Δ*arrR* strain represented an *A. veronii* knockout strain lacking the full-length *arrR* gene. Consequently, Δ*arrS*Δ*arrR* represented a corresponding double knockout strain. Unless otherwise stated, all strains were cultured on Luria Bertani cluture (LB), maintaining a temperature of 30°C for *A. veronii* and 37°C for *E. coli*. Antibiotic was used based on the specific plasmid carried by each strain, with final concentrations as follows: ampicillin (Amp) at 50 μg/mL, chloramphenicol (Cm) at 25 μg/mL, kanamycin (Km) at 50 μg/mL, and tetracycline (Tc) at 12.5 μg/mL.

### Construction of recombinant plasmids

Plasmids employed in constructing recombinant vectors were listed in Supplementary Table S1, and all utilized primers were provided in Supplementary Table S2. In this study, two methods were utilized for the construction of recombinant vectors: the conventional method involved the use of restriction endonucleases and T4 DNA ligase, while the other one utilized an Ultra One Step Cloning Kit (Vazyme, China) that achieved directional cloning through the recombinase-mediated recombination. The latter was used for the insertion of multiple fragments into the plasmids.

Using the genomic DNA of *A. veronii* WT as template, the *arrS* promoter (P*_arrS_*) was cloned using primers P*_arrS_*-F/R to construct pBXcmT-P*_arrS_*; the HPT domain of ArrS was cloned using primers pTRGHPT-F/R to construct pTRG-HPT; the REC domain of ArrR was cloned using primers pBTREC-F/R to construct pBT-REC; the ArrS^820-1414^ was cloned using primers pETArrS-F/R to construct pET28a-ArrS; the ArrR was cloned using primers pETArrR-F/R to construct pET28a-ArrR. Using the genomic DNA of *P. aeruginosa* as template, the *dgcH* was cloned using primers DgcH-F/R to construct pBBR-*dgcH*.

To obtain pET28a-ArrRm, point-mutant PCR was performed on pET28a-ArrR. The phosphorylation site of the REC domain was mutated to alanine (D59A) using primers ArrRm-F/R, followed by mutation of the Mg^2+^ binding site (E14A, D15A) using primers ArrRm2-F/R. To obtain pBXcmT-P*_arrS_* m, point-mutant PCR was performed on pBXcmT-P*_arrS_* using primers AGRm-F/R to introduce mutations at its ARG-BOX. To obtain pBBR-ArrR^aal^, point-mutant PCR was performed on pBBR-ArrR using primers AAL-F/R, and the critical glutamate of EAL domain was mutated to alanine (E176A). To obtain pBBR-ArrRm, the mutated *arrR* was cloned from pET28a-ArrRm using primers pBBR*arrR*-F/R and ligated together with P*_arrS_*.

### Construction of the knockout and complementary strains

The allele-exchange vector pRE112 was utilized for *arrS* and *arrR* knockout through homologous recombination. To induce genetic recombination, the upstream and downstream homologous arms flanking the target fragment should be positioned in close proximity to each other. In pRE112-*arrS*, homologous arms flanking the *arrS* gene segment (from 271 to 668 bp) were cloned using primers *arrS*arm1-F/R and *arrS*arm2-F/R, respectively; whereas in pRE112-*arrR*, homologous arms flanking the whole *arrR* gene were cloned using primers *arrR*arm1-F/R and *arrR*arm2-F/R. Under the help of the donor strain *E. coli* WM3064, pRE112-*arrS* and pRE112-*arrR* were transformed into *A. veronii* via bacterial conjugation. Subsequently, *A. veronii* carrying pRE112-*arrS* or pRE112-*arrR* was cultured overnight in non-antibiotic LB for the first recombination, and then grow on LB plates containing 22% sucrose and 25 μg/μL Amp for the second recombination. Knockout strains were verified by colony PCR using primers pairs *arrS*-F0/R0 for Δ*arrS* and *arrR*-F0/R0 for Δ*arrR*, followed by confirmation of sequencing analysis.

The broad-host-range shuttle vector pBBR1MCS-2 was employed for the construction of *arrS* or *arrR* complementation strains. For pBBR-*arrS*, the full-length *arrS* gene and its promoter were cloned from the *A. veronii* genome using primers pBBR*arrS*-F/R; For pBBR-*arrR*, the full-length *arrR* gene and P*_arrS_* were cloned and ligated adjacently using primers pBBRP*_arrS_*-F/R and pBBR*arrR*-F/R. Subsequently, Δ*arrS* with pBBR-*arrS* and Δ*arrR* with pBBR-*arrR* were obtained through bacterial conjugation, serving as the complementation strains for Δ*arrS* and Δ*arrR*, respectively.

### Protein expression and purification

The expressions of ArrS^820-1414^, ArrR, ArrRm, and ArgR were induced in *E. coli* BL21 (DE3) at 16°C. Subsequent to induction, the cells were harvested using a 50 mM pre-cooled Tris–HCl buffer (pH 8.0) and subjected to sonication on ice for 15 min. Following centrifugation at 4°C, the resulting crude protein extracts underwent purification through Ni-NTA agarose gel, employing affinity chromatography. ArrS^820-1414^ and ArgR were eluted using a 250 mM imidazole buffer, whereas ArrR and ArrRm were eluted using a 150 mM imidazole buffer. The eluted solution, enriched with the target proteins, was screened with G-250, dialyzed in a solution containing 50 mM Tris–HCl and 20% glycerol at pH 8.0, partitioned into 2 mL centrifuge tubes, and subsequently stored at −80°C.

### Phosphorylation and phosphotransfer

ArrS^820-1414^, ArrR, and ArrRm underwent phosphorylation in a phosphorylation buffer composed of 50 mM Tris–HCl, 100 mM NaCl, 5 mM MgCl_2_, 2 mM Dithiothreitol, at a pH of 8.0. For the autophosphorylation, 5 mg of ArrS^820-1414^ or ArrR were separately incubated with 100 μM ATP in 50 μL of phosphorylation buffer at room temperature for 30 min. For the phosphotransfer, additional 1 mg of ArrS^820-1414^ was added.

### Detection of the phosphodiesterase activity

The procedure for detecting PDE activity was as previously outlined by Bobrov and He Chenyang (Bobrov et al., 2005; Yang et al., 2012). In this investigation, 200 ng each of ArrS^820-1414^, ArrR, or ArrRm were individually incubated with 10 mM bis (4-nitrophenyl) phosphate (BNPP) in the PDE buffer (50 mM Tris–HCl, 1 mM MnCl_2_, pH 8.5) at 30°C. The PDE activity was evaluated based on the release rate of p-nitrophenol in the reaction mixtures, quantified by measuring OD_410_.

### Quantification of intracellular c-di-GMP levels

The fluorescence reporter vector pBBR-P*_cdrA_*-GFP was constructed to quantify the intracellular c-di-GMP concentrations. In this construct, the promoter region of *cdrA* from *P. aeruginosa* (from −290 to +12 bp) was placed upstream of the GFP gene. The interaction of c-di-GMP with FleQ (encoded by ONR73_15360 in *A. veronii*) would lead to transcriptional derepression of P*_cdrA_*, thereby enabling enhanced transcriptional activity in response to c-di-GMP binding (Rybtke et al., 2012). Thus, pBBR-P*_cdrA_*-GFP established a direct proportional relationship between fluorescence intensity and intracellular c-di-GMP levels. Intracellular c-di-GMP concentrations can be quantified by measuring fluorescence intensity at an excitation wavelength of 479 nm and an emission wavelength of 525 nm. Furthermore, liquid chromatography coupled with tandem mass spectrometry (LC–MS) was employed as well to quantify the intracellular c-di-GMP levels, as detailed by Bahre and Kaever (2017). 5 mL of bacteria were collected and the c-di-GMP was extracted by 1 mL of extraction solution (40% methanol, 40% acetonitrile, 20% water). The protein precipitates were stored at −20°C for total protein quantification, while the supernatant was transferred into a 10 mL centrifuge tube for lyophilization and redissolution. These samples were compared against a standard curve derived from measurements of c-di-GMP standard substances with known concentrations (Merck, China). The intracellular c-di-GMP levels were then normalized against total protein contents and expressed as pmol of c-di-GMP/mg of total protein.

### Bacteria two-hybrid and one-hybrid assays

The bacterial two-hybrid system (Joung et al., 2000) was employed to investigate the interaction between ArrS and ArrR. The HPT domain of ArrS was fused with RNA polymerase alpha subunit (RNAPα) in the pTRG-HPT plasmid, whereas the REC domain of ArrR was fused with λcI in the pBT-REC plasmid. These plasmids were co-transformed into *E. coli* XL1-Blue MRF′, followed by overnight culture in M9^+^ His-dropout broth, gradient dilution, and growth on dual-selective solid medium with 10 mM 3-aminotriazol (3-AT) and 12.5 μg/mL streptomycin. The co-transformed strain carrying pBT-LGF2 and pTRG-Gal11^p^ served as the positive controls.

The bacterial one-hybrid system (Guo et al., 2009) was utilized to verify the interaction between ArgR and P*_arrS_*. The plasmid pTRG-ArgR expresses a fusion protein of ArgR and RNAPα, while the plasmid pBXcmT-P*_arrS_* has the promoter P*_arrS_* (from −132 to +3 bp) inserted before the *HIS3-aadA* reporter. Moreover, point mutation PCR was conducted on pBXcmT-P*_arrS_* to introduce a 2-bp point mutation in the ARG-BOX of P*_arrS_*, resulting in pBXcmT-P*_arrS_* m. The co-transformed strain of pBXcmT-P*_arrS_* and pTRG-SmpB was utilized as a positive control (Liu et al., 2015).

### Chromatin immunoprecipitation followed by sequencing (ChIP-seq)

ChIP-seq was performed as previously described by Wang et al. (2024). The WT strain carrying pBBR-*argR* was cultured in M9 medium, collected by centrifugation, and cross-linked with 1% formaldehyde. The cross-link was quenched with 125 mM glycine. Subsequently, the bacterial cells were harvested by centrifugation, washed with pre-cooled PBS buffer, preserved on dry ice, and dispatched to SEQHEALTH (Wuhan, China) for ChIP-seq analysis to identify the target genes of ArgR. FLAG antibodies were employed for the immunoprecipitation of DNA-protein complexes. The raw data were subjected to quality assessment using FastQC (v0.11.5) and subsequently refined using Trimmomatic (v 0.39) before being mapped to the genome of WT. ArgR peaks were generated and visualized using MACS2 (v2.1.2) and the IGV Viewer (v 2.11.2), respectively.

### Electrophoretic mobility shift (EMSA) assay

The primers P*_arrS_*-R was labeled with 6-Carboxyfluorescein (6-FAM) at its 5′ end, and P*_arrS_*-F and P*_arrS_*-R were used to amplified the luciferase-modified P*_arrS_*, as well as its mutated ARG-BOX variant, P*_arrS_* m. For EMSA, various concentrations of P*_arrS_* or P*_arrS_* m were separately incubated with ArgR in EMSA buffer (50 mM Tris–HCl, 5 mM MgCl_2_, 1 mM dithiothreitol) at room temperature. Then the reaction mixtures were subjected to electrophoresis on a 6% polyacrylamide gel using Tris-Borate-EDTA buffer. Following 2 h of electrophoresis at 4°C, the migration of P*_arrS_* or P*_arrS_* m was visualized using a biomolecular imager Typhoon FLA9500 (GE Healthcare, America).

### Biofilm detection

Biofilm formation was assessed using the crystal violet method, performed in 96-well microtiter plates (Coffey and Anderson, 2014). 100 μL of the bacterial suspension at a final OD_600_ of 0.1 was aliquoted into separate wells in a 96-well PVC plate and incubated in a stationary position for 24 h. After incubation, the plate was washed three times and treated with 150 μL methanol for 20 min, followed by air-drying and staining with 150 μL of 0.5% crystal violet for an additional 20 min. Excess crystal violet was removed by flowing water, and 150 μL of 33% acetic acid was added to each well after drying. Following a 30-min incubation at 37°C, biofilm formation was quantified by measuring the OD_590_.

### Motility detection

The overnight-cultured strains were washed, resuspended in PBS buffer, and diluted to an OD_600_ of 2. For swimming, 2.5 μL of the aforementioned bacterial solution was spotted onto swimming semi-solid plates (composed of 1% peptone, 0.5% NaCl, 0.25% agar). After culturing for 10 h, these plates were further incubated at 4°C for an additional 12 h to enhance the visibility of the swimming zone. The swimming radii were then measured. For swarming, an additional 2.5 μL of the bacterial solution was spotted onto swarming semi-solid plates (consisting of 1% peptone, 0.5% NaCl, 0.35% agar), and the swarming radii were measured following a 16-h incubation.

### Reverse transcription quantitative polymerase chain reaction (RT-qPCR)

*Aeromonas veronii* WT and Δ*arrS* were cultured in LB for 21 h with an initial optical density (OD_600_) of 0.01. The bacteria were then collected by centrifugation, and total RNA was extracted using the Bacteria Total RNA Isolation Kit (Sangon, Shanghai), and reverse transcription was then performed using the HiScript II Q RT SuperMix (Vazyme, Nanjing). Subsequently, qPCR system was configured with the ChamQ SYBR Color qPCR Master Mix (Vazyme, Nanjing). RT-qPCR was performed on the Recho LightCycler 96 using a two-step method. The relative transcription level was calculated using the 2^−ΔΔCT^ method, with the DNA helicase *gyrB* as the reference gene.

### Proteomic analysis

After overnight culture in LB, the activated *A. veronii* WT and Δ*arrS* were inoculated into fresh LB at an optical density (OD_600_) of 0.01, and continued to be cultured at 150 rpm for 18 h. Subsequently, WT and Δ*arrS* were harvested by centrifugation, frozen in liquid nitrogen, stored on dry ice, and sent to APTBIO (Shanghai, China) for label-free quantitative proteomics. The technical workflow encompasses protein extraction, protein quantification, enzymolysis, LC–MS/MS, database searching, and bioinformatics analysis. The acquired raw data were processed using MaxQuant (v1.6.2.0) for database searching and quantitative analysis. The Gene Ontology annotation of all differentially expressed proteins was performed using Blast2GO (v 5.2.5) (Gotz et al., 2008).

### Ultraviolet radiation detection

The overnight-cultured *A. veronii* WT, Δ*arrS* and Δ*arrS* + pBBR-*arrS* were collected, resuspended in PBS buffer, and diluted to a concentration of 10^7^ CFU/mL. Then 10 mL of the bacterial suspension was poured onto a 90 mm plate and exposed to ultraviolet (UV) radiation for 60 s using a UV-light cross-linker SCIENTZ 03-II (SCIENTZ, China), with a wavelength of 254 nm and power intensity of 4 mW/cm^2^. 100 μL of the irradiated bacterial solution was then applied onto LB plates and incubated in darkness for 24 h. The colony-forming units (CFUs) on the plate were counted to determine the survival rate.

### Toxicity challenge in mice

Kunming mice were purchased from Hunan SJA Laboratory Animal Co., Ltd., and all procedures followed the Hainan University Guidelines for the Care and Use of Laboratory Animals, approved by the Animal Ethics Committee of Hainan University. Five-week-old mice were allocated into four groups, each consisting of six individuals. Three of them received intraperitoneal injections of WT, Δ*arrS* and Δ*arrS* + pBBR-*arrS* at a dose of 10^5^ CFU/g, while the remaining group was injected with 100 μL PBS buffer as a control. Then, the mice fasted for 24 h and their survival rate was monitored for 7 days. Simultaneously, four additional groups of mice underwent intraperitoneal injection as well, and their orbital blood was collected 12 h later. The quantifications of TNF-α, IFN-γ, IL-1β, IL-4, and IL-6 in serum were determined by an ELISA kit (Meibiao Biotechnology, Jiangsu). After blood collection, the mice were immediately dissected, and their spleen, kidney, and liver were washed and weighed. A portion of these organs was homogenized using a tissue crusher, followed by dilution and plating on LB plates with ampicillin for the quantification of *A. veronii* burdens. Another portion was treated with 4% paraformaldehyde and prepared for paraffin sections stained with hematoxylin–eosin (HE), ultimately undergoing pathological observation under a microscope.

### Statistical analysis

Statistical analysis and graphical representation were carried out using GraphPad Prism v8.0.2 (GraphPad, America), with a minimum of three replicates per experiment. Significance was assessed using multiple t-tests, with error bars representing standard deviation (SD). The symbol “*” denoted significant difference (*p* < 0.05) and “**” denoted extremely significant difference (*p* < 0.01), while “ns” denoted no significant difference.

## Results

### ArrS and ArrR constitute a TCS

**Figure 1:**
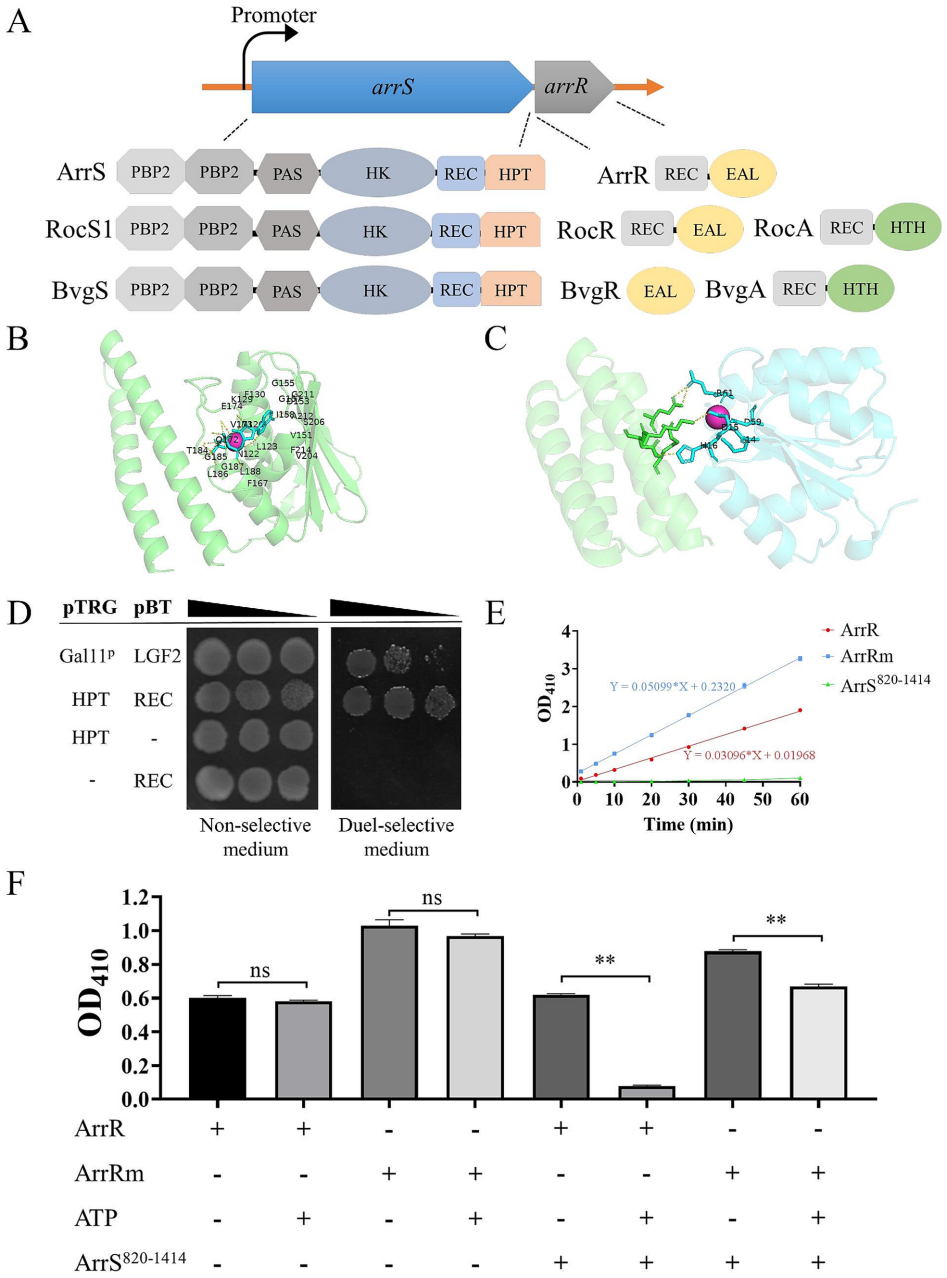
Formation of a functional TCS by ArrS and ArrR. **(A)** Comparative structural analysis of ArrS, ArrR and their homologous proteins. **(B)** Autophosphorylation model. The green region indicates the HPT domain of ArrS, the red spheres represent Mg^2+^, and the blue region represents ATP. Residue numbering begins at the first residue of the HPT domain. **(C)** Interaction model. The green region represents the HPT domain of ArrS, the blue region represents the REC domain of ArrR, and the red spheres represent Mg^2+^. **(D)** Confirmation of the interaction between the HPT domain and REC domain via two-hybrid. The co-transformed strain containing pBT-LGF2 and pTRG-Gal11^p^ was used as the positive control. **(E)** Assessment of the PDE Activity. Using 10 mM BNPP as the substrate, PDE activity were quantified by measuring the release rate of p-nitrophenol (OD_410_). **(F)** Investigation of the PDE activity and phosphotransfer. ArrR or ArrRm were incubated with ArrS, ATP or both in PDE buffer for 20 min. Experiments were replicated three times in panels **(E,F)**. *p*-values were obtained from t-tests and the error bars indicate the SD. **denoted extremely significant difference (*P*<0.01).

The conserved domains of ArrS and ArrR were analyzed by CD-search (Lu et al., 2020), and the results were presented in Figure 1A. ArrS exhibited common domains with BvgS from *Bordetella pertussis* and RocS1 from *Pseudomonas aeruginosa*. All of them consisted of two type-2 periplasmic-binding fold protein (PBP2) domains at periplasmic region, followed by a PAS domain, an HK domain, a REC domain, an HPT domain in the cytoplasmic region. Notably, ArrR shared a similar structural composition with RocR, both of them encompassed a REC domain and an EAL domain. The same domains suggested ArrS and ArrR might form a functional TCS like RocS/RocR. Additionally, as depicted in Supplementary Figures S1, S2, *arrS* and *arrR* were co-transcribed and were observed across at least 15 *Aeromonas*, including the most common clinical species (*A. caviae*, *A. dhakensis*, *A. veronii*, *A. hydrophila*). These discoveries reinforced our argument. But in contrast to their homologous TCS counterparts in *B. pertussis* and *P. aeruginosa*, in the proximity of ArrS/ArrR there was no gene similar to *bvgA* or *rocA* that encoding a transcription factor, indicated ArrS and ArrR constituted a distinctive TCS.

To establish the existence of a novel TCS consisting of ArrS and ArrR, we initially employed AlphaFold 3 to simulate the ATP binding ability of ArrS and its interaction with ArrR. The pLDDT scores and the PAE plots of above models were shown in Supplementary Figure S3. The autophosphorylation model (Figure 1B) showed that ATP and Mg^2+^ were encapsulated within the pocket of the HK domain, with ipTM and pTM scores of 0.96 and 0.83, respectively. The residues predicted to interact with ATP and Mg^2+^ in this model were consistent with those identified by CD-search, strongly indicating that ArrS possesses autophosphorylation activity. The interaction model (Figure 1C) showed that the HPT domain of ArrS interacted with the REC domain of ArrR in the presence of Mg^2+^, with ipTM and pTM scores of 0.8 and 0.87, respectively. Once again, the AlphaFold 3 model and the CD search analysis were consistent. The key residues annotated by the CD-search were shown in Supplementary Figure S4. In conclusion, the models predicted by AlphaFold 3 strongly support the possibility that ArrS and ArrR form a TCS.

Subsequently, we examined the interaction between ArrS and ArrR using a bacteria two-hybrid system. In the study of RocR and RocS1, the HPT domain and the REC domain were used for detecting the interaction between SK and RR (Kulasekara et al., 2005). Consequently, we cloned the HPT domain of ArrS into the pTRG plasmid and the REC domain of ArrR into the pBT plasmid. As depicted in Figure 1D, the co-transformated strains carrying pTRG-HPT and pBT-REC exhibited enhanced growth in dual-selective medium compared to the positive control containing pBT-LGF2 and pTRG-Gal11^p^. This observation demonstrated a robust interaction between the HPT domain and REC domain, confirming the binding of ArrS to ArrR.

Generally, SK regulates the active state of RR through phosphotransfer. Thus, we verified the occurrence of phosphotransfer by examining the effect of ArrS on the PDE activity of ArrR. As shown in Supplementary Figure S5, we expressed and purified the truncated ArrS, the full-length ArrR, and the mutated ArrR in *E. coli* BL21 (DE3). The truncated ArrS, denoted as ArrS^820-1414^, omitted the PBP2 and PAS domains at C-terminus while retaining the domains responsible for autophosphorylation and phosphotransfer, which is more suitable for expression and purification. The mutated ArrR, denoted as ArrRm, harbored point mutations at G_14_, D_15_ and D_59_ that are critical for REC domain (Supplementary Figure S4), rendering it theoretically unresponsive to accept phosphate from ArrS. Firstly, we verified the PDE activity of ArrR and ArrRm using BNPP as the substrate. As shown in Figure 1E, BNPP continued to decompose in the presence of ArrR, confirming the PDE activity of ArrR; the decomposition rate of BNPP treated with ArrRm was slightly but significantly higher than that of ArrR (approximately 1.65 times), indicating that the non-phosphorylated form of ArrR possesses strong PDE activity. Subsequently, we investigated the PDE activity of ArrR and ArrRm when incubated with ArrS^820-1414^, ATP, or both. As illustrated in Figure 1D, ATP alone did not significantly influence the PDE activity of ArrR or ArrRm, nor did ArrS. However, after incubation with both ArrS^820-1414^ and ATP for 20 min as shown in Figure 1F, the PDE activity of ArrR decreased to 12.57%, while ArrRm retained 76.25% of its PDE activity under identical condition. The impact exerted by ArrS^820-1414^ and ATP on ArrRm was significantly lower than that observed for ArrR. This phenomenon suggested that the PDE activity of ArrR was inhibited by ArrS-mediated phosphorylation, along with the occurrence of phosphotransfer from ArrS to ArrR.

### ArrS and ArrR alter the intracellular c-di-GMP concentration

Motility and biofilm formation represent two prominent phenotypes in bacteria that are influenced by the intracellular c-di-GMP levels. Thus, we conducted an initial assessment of the influence of ArrS on intracellular c-di-GMP levels by assessing motility and biofilm formation. As illustrated in Supplementary Figure S6, we generated the *A. veronii arrS* knockout strain Δ*arrS*, and deletion of *arrS* demonstrated no discernible impact on the growth in *A. veronii*. Furthermore, Δ*arrS* + pBBR-*arrS* and WT + pBBR-*dgcH* were generated as well and served as controls. DgcH is a guanylate cyclase from *P. aeruginosa* and its expression will lead to an elevated c-di-GMP levels in cells (Liu et al., 2023).

**Figure 2:**
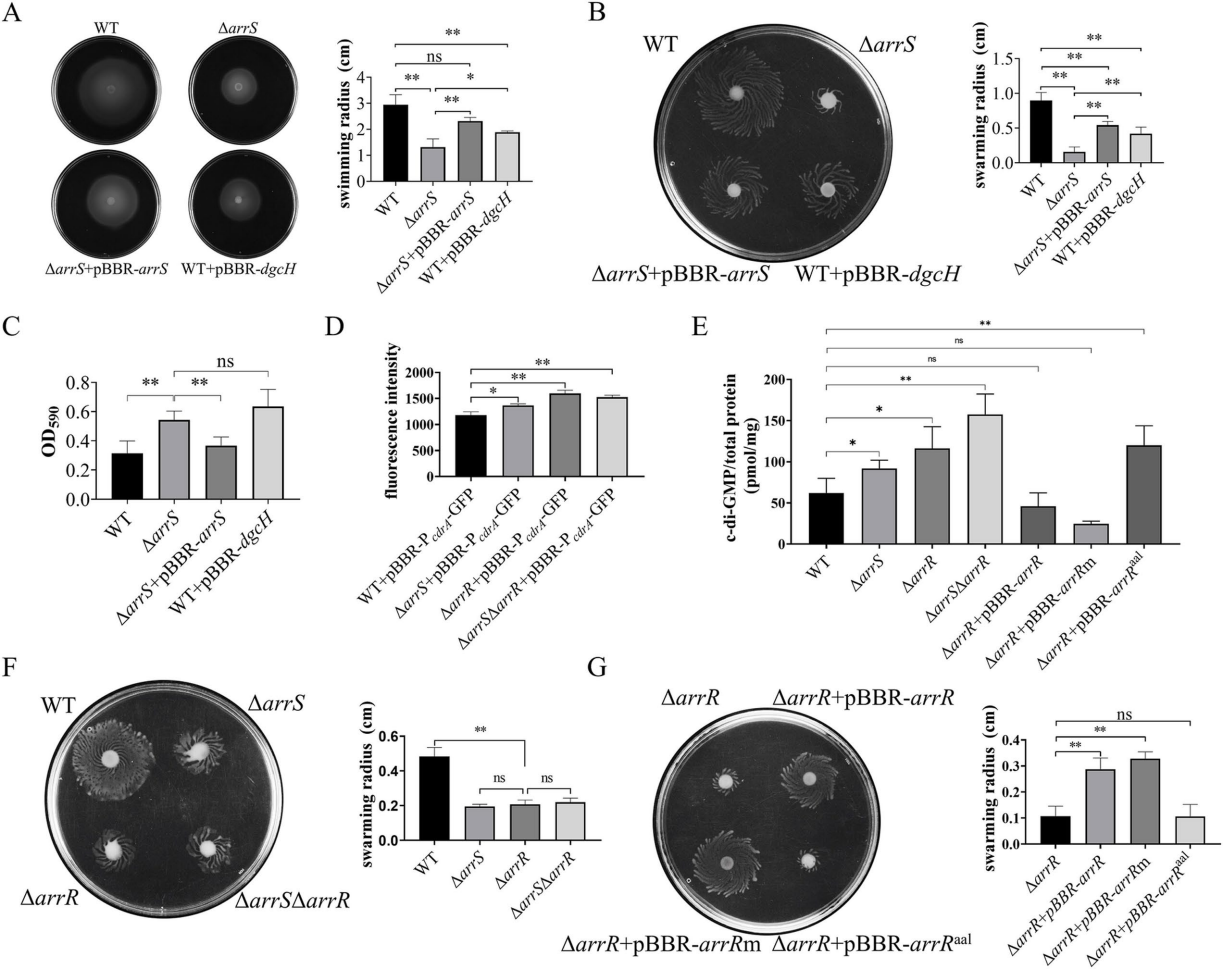
Regulation of c-di-GMP mediated by ArrS and ArrR. **(A)** Evaluation of the influence of ArrS on swimming ability. **(B)** Assessment of the effect of ArrS on swarming ability. **(C)** Detection of the impact of ArrS on biofilm formation. Biofilm quantity was assessed by measuring the intensity of crystal violet (OD_590_). **(D)** Measurement of the intracellular c-di-GMP levels using pBBR-P*_cdrA_*-GFP. The fluorescence intensity was directly correlating with intracellular c-di-GMP levels. **(E)** Quantification of the intracellular c-di-GMP levels using LC–MS. **(F)** Verification of the swarming ability for knockout strains and **(G)** complementary strains. Experiments were replicated three times in panels **(A,D)**, while four times in panels **(B,E,F,G)**, and five times in panel **(C)**. *p*-values were obtained from t-tests and the error bars indicate the SD.

The motility findings are illustrated in Figures 2A,B. The mean swimming radius for Δ*arrS* was 1.32 cm, whereas the mean swimming radii for WT and Δ*arrS* + pBBR-*arrS* were 2.23-fold and 1.76-fold greater than that of Δ*arrS*, respectively. Similarly, the swarming radii exhibited comparable trends. The mean swarming radius for Δ*arrS* was 0.158 cm, while the mean swarming radii for WT and Δ*arrS* + pBBR-*arrS* were 5.70-fold and 3.43-fold greater, respectively. For WT + pBBR-*dgcH*, the average swimming and swarming radii were 1.89 cm and 0.418 cm, respectively, falling within the range observed for Δ*arrS* and Δ*arrS* + pBBR-*arrS*. Furthermore, as depicted in Figure 2C, the amount of biofilm generated by WT and Δ*arrS* + pBBR-*arrS* was determined to be 57.8 and 67.5% of that produced by Δ*arrS*, respectively. In contrast, WT + pBBR-*dgcH* exhibited a slightly higher amount of biofilm compared to Δ*arrS*. Δ*arrS* exhibited enhanced biofilm formation but weaker motility, which is consistent with previous observations of *A. veronii* under elevated c-di-GMP concentrations (Rahman et al., 2007; Robinson et al., 2021). Additionally, the phenotypic similarities between WT + pBBR-*dgcH* and Δ*arrS* provide further evidence suggesting that these observations should be attributed to an increased concentration of c-di-GMP *in vivo*.

To prove this speculation, we constructed a fluorescence reporter vectors, namely pBBR-P*_cdrA_*-GFP, to assess the intracellular c-di-GMP levels in *A. veronii*. In *P. aeruginosa*, the FleQ protein binds to the promoter region of the *cdrA* gene and inhibits its transcription, while the binding of c-di-GMP to FleQ alleviates this inhibitory effect (Rybtke et al., 2012). Consequently, the fluorescence intensity observed in *A. veronii* with pBBR-P*_cdrA_*-GFP should be directly proportional to the intracellular c-di-GMP concentration. As depicted in Figure 2D, the mean fluorescence value of pBBR-P*_cdrA_*-GFP in WT was 1178.3, significantly lower than that of Δ*arrS*, with a mean fluorescence value of 1366.7. This result reconfirmed that the intracellular c-di-GMP in WT was significantly lower than that in Δ*arrS*.

Elevated c-di-GMP levels in Δ*arrS* were unexpected, given its capacity to inhibit the PDE activity of ArrR. Consequently, we further generated *A. veronii* knockout strains Δ*arrR* and Δ*arrS*Δ*arrR*, followed by a quantitative analysis of intracellular c-di-GMP levels using the pBBR-P*_cdrA_*-GFP and the LC–MS. As depicted in Figure 2D, the fluorescence intensity was 1596.8 in Δ*arrR* and 1525.0 in Δ*arrS*Δ*arrR*, both significantly higher than that in WT. In alignment with the aforementioned findings, LC–MS analysis (Figure 2E) revealed that the intracellular c-di-GMP concentrations was 91.86 pmol/mg in Δ*arrS*, 116.17 pmol/mg in Δ*arrR*, and 157.54 pmol/mg in Δ*arrS*Δ*arrR*. All of them were significantly higher than that in WT (62.00 pmol/mg). This finding corroborated again that the deletion of *arrS* indeed led to an increase in intracellular c-di-GMP observed *in vitro*, indicating ArrS might have an alternative mechanism besides phosphorylating ArrR. Furthermore, although the c-di-GMP concentration in Δ*arrS*Δ*arrR* was higher than Δ*arrR*, no statistically significant difference was observed between the c-di-GMP concentrations in Δ*arrS*Δ*arrR* and Δ*arrR*. Consistently, as shown in Figure 2F, no significant difference was observed in the swarming radii of Δ*arrS*, Δ*arrR*, and Δ*arrS*Δ*arrR*. This indicated that ArrS and ArrR should achieve their functions in a common mechanism, which is a two-component system.

To demonstrate the observed intracellular c-di-GMP fluctuation was attributed to the PDE activity of ArrR, we constructed complementation strains for Δ*arrR* using original ArrR, phosphorylation-insensitive ArrRm and PDE-inactivated ArrR^aal^, respectively. As depicted in Figure 2E, the c-di-GMP content was measured as 46.01 pmol/mg for Δ*arrR* + pBBR-*arrR*, 24.55 pmol/mg for Δ*arrR* + pBBR-*arrR*m, and 120.01 pmol/mg for Δ*arrR* + pBBR-*arrR*^aal^. The observed alterations were consistent with the expected PDE activity of ArrR, ArrRm and ArrR^aal^. Complementation with ArrR or ArrRm significantly reduce the intracellular c-di-GMP levels, whereas complementation of ArrR^aal^ lacking PDE activity had minimal impact. Similarly, these strains exhibited comparable performance in swarming ability, as illustrated in Figure 2G. Furthermore, a consistent trend was observed in both the biofilm formation and swimming motility (Supplementary Figure S7). In conclusion, above findings supported our hypothesis that the PDE activity of ArrR modulates the intracellular c-di-GMP levels of *A. veronii*.

### ArgR functions as the transcriptional repressor of *arrS*

**Figure 3:**
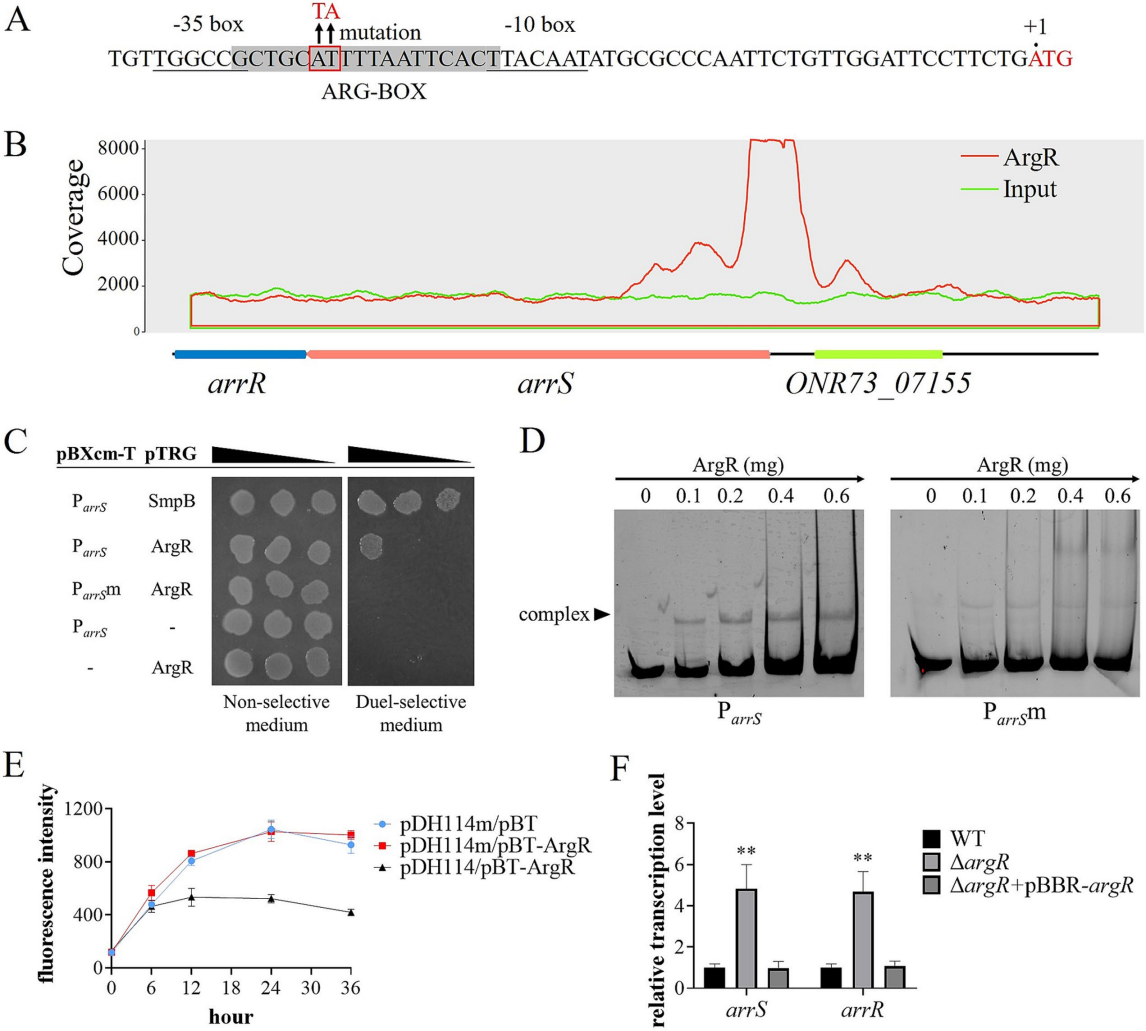
ArgR as a transcriptional repressor of *arrSR*. **(A)** P*_arrS_* and its mutation sites at ARG-BOX. ARG-BOX was highlighted in gray, −10 and −35 regions were demarcated by underline, and the mutation site was located within the red frame. **(B)** ChIP-seq revealed ArgR binding to P*_arrS_*. The peaks of ArgR and input were depicted by red and blue curves respectively, with the corresponding genomic positions illustrated below. **(C)** Verification of ArgR binding to P*_arrS_* via one-hybrid. The mutation sites of P*_arrS_* m were shown in panel **(A)**. **(D)** Verification of ArgR binding to P*_arrS_* via EMSA. P*_arrS_* and P*_arrS_* m were labeled with 6-FAM and separately incubated with varying concentrations of ArgR. **(E)** Validation of the transcriptional function of ArgR via fluorescent reporter. The expression of GFP was regulated by P*_arrS_* in pDH114 and P*_arrS_* m in pDH114m, while pBT-ArgR was used to express ArgR. **(F)** Confirmation of ArgR inhibition on *arrSR* via RT-qPCR. Experiments in panels **(E,F)** were conducted with five replicates. *p*-values were obtained from t-tests and the error bars indicate the SD. **denoted extremely significant difference (*P*<0.01).

The sequence analysis of P*_arrS_* revealed an ARG-BOX (Cho et al., 2015) located between the −10 and −35 regions, as depicted in Figure 3A. The ARG-BOX serves as the binding site for the global transcription factor ArgR, which plays a crucial role in regulating arginine metabolism and transport. Additionally, the results of ChIP-Seq indicated a substantial enrichment of P*_arrS_* by ArgR (Figure 3B), with a pileup ratio 22.86 times that of the input. These findings prompted our suspicion of a direct interaction between ArgR and P*_arrS_*. Thus, the bacterial one-hybrid was conducted and the results were shown in Figure 3C, where *E. coli* XL1-Blue MRF′ carrying pBXcmT-P*_arrS_* and pTRG-ArgR exhibited robust growth on the dual-selective medium. Furthermore, as illustrated in Figure 3D, P*_arrS_* labeled with 6-FAM was retained in the EMSA assay following incubation with ArgR. When a point mutation illustrated in Figure 3A was introduced into the ARG-BOX of P*_arrS_*, XL1-Blue MRF′ carrying pBXcmT-P*_arrS_* m and pTRG-ArgR failed to grow on the dual-selective medium (Figure 3C) and P*_arrS_* m did not exhibit retention during gel electrophoresis (Figure 3D). These evidences demonstrated the binding ability of ArgR to P*_arrS_* and the dependence of ArgR binding on ARG-BOX.

To investigate the effects of ArgR binding on P*_arrS_*, we used a fluorescence plasmid, pDH114, which expresses GFP under the control of P*_arrS_* (Liu et al., 2015). Furthermore, we constructed pDH114m, a mutant version that incorporated mutations in its ARG-BOX while maintaining its promoter activity (Supplementary Figure S8). The fluorescence intensity of *E. coli* XL1-Blue MRF′ carrying pDH114 or pDH114m was detected in the presence or absence of ArgR. As depicted in Figure 3E, when ArgR was expressed by the pBT-ArgR plasmid, there was a significant reduction in the fluorescence intensity of host cells carrying pDH114. However, for pDH114m, no change was observed upon ArgR expression. These results suggested that ArgR inhibited the transcription of P*_arrS_*. Moreover, the reverse transcription quantitative polymerase chain reaction (RT-qPCR) analysis yielded similar results, as exhibited in Figure 3F. The transcription levels of both *arrS* and *arrR* in Δ*argR* was significantly higher than those observed in WT and Δ*argR* + pBBR-*argR*, indicating that the knockout of ArgR alleviated its restriction on *arrS* transcription.

### ArrS modulates the virulence of *Aeromonas veronii*

**Figure 4:**
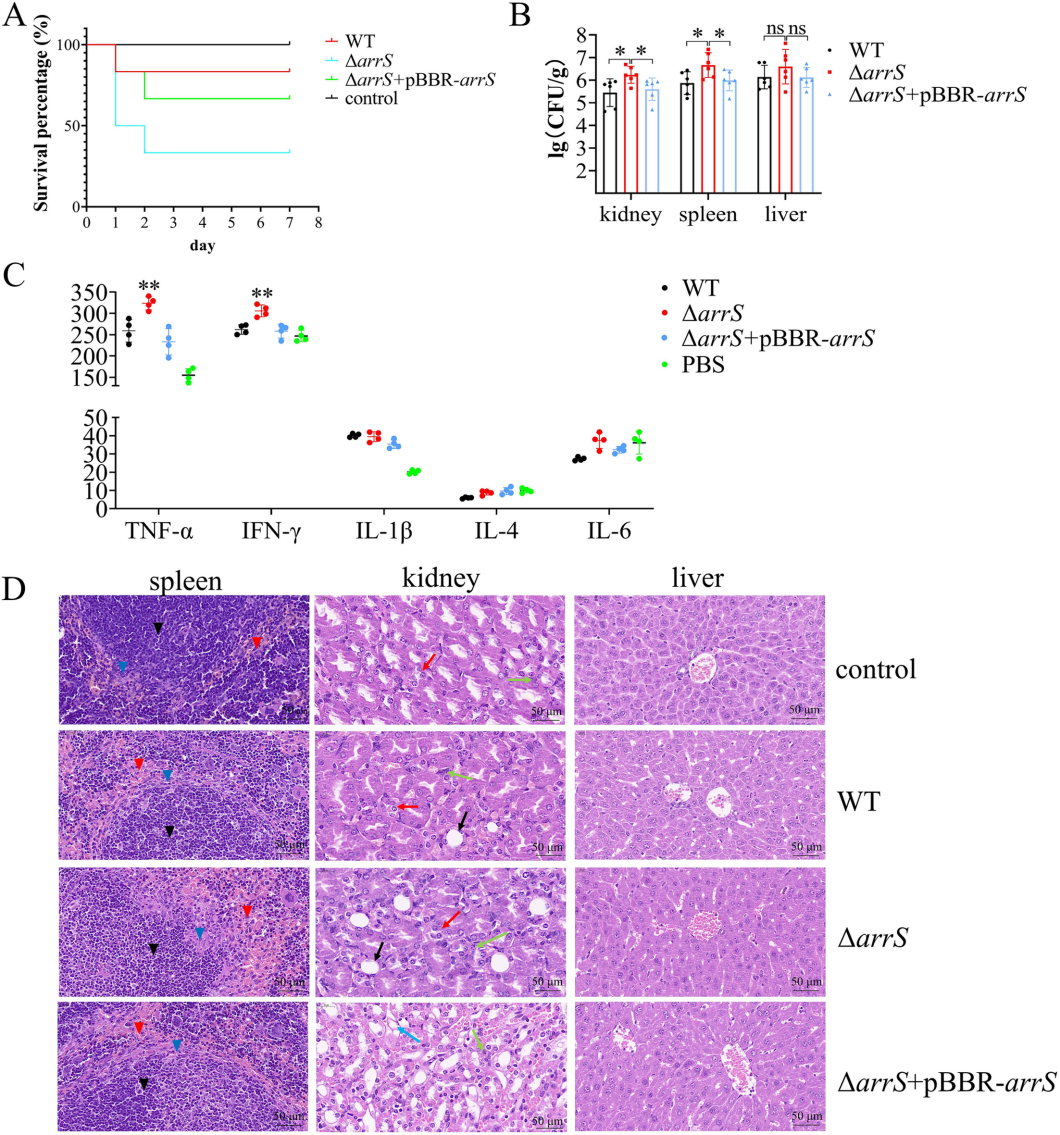
Involvement of ArrS in the pathogenicity of *A. veronii*. **(A)** Survival curve of mice after infection. **(B)** Assessment of bacterial counts in kidney, spleen and liver. **(C)** Determination of immune factors via ELISA. **(D)** Pathological observation of kidney, spleen and liver. Pathological changes were observed with a scale of 50 μm using paraffin sectioning and HE staining. The black, blue, and red inverted triangles represent the white pulp, marginal zone, and red pulp of the spleen, respectively. The black, red, green, and blue arrows represent the renal tubular epithelial cell necrosis, renal tubular atrophy, inflammatory cell infiltration, and vacuolar degeneration of renal tubules of the kidney, respectively. The Experiments were conducted with six replicates in panel **(B)** and four replicates in panel **(C)**. *p*-values were obtained from t-tests and the error bars indicate the SD. *denoted significant difference (*P*<0.05) and **denoted extremely significant difference (*P*<0.01).

TCSs associated with c-di-GMP typically play a crucial role in the regulation of virulence in bacteria, such as BvgS from *B. pertussis* and RocS1 from *P. aeruginosa* (Dupre et al., 2015a; Sultan et al., 2021). It was postulated that ArrS/ArrR might play a role in the regulation of virulence as well. Therefore, we performed a toxicity assessment of *A. veronii* in mice. The survival rates within 7 days were recorded and depicted in Figure 4A. At the end of this duration, five mice in the WT group and four mice in the Δ*arrS* + pBBR-*arrS* group survived; however, only two mice survived in the Δ*arrS* group. According to the Mantel-Cox analysis, at an injected dose of 10^5^ CFU/g, the survival rate of mice in the Δ*arrS* group was significantly lower than the control group (*p* = 0.0178). In contrast, the WT and the Δ*arrS* + pBBR-*arrS* groups did not differ significantly from the control. These results indicated that the knockout of *arrS* enhanced the pathogenicity of *A. veronii*.

Subsequently, we quantified the numbers of *A. veronii* in the spleen, liver, and kidney, analyzed the relative levels of immune factors in serum, and conducted hematoxylin–eosin staining and histological examination on these three organs. As depicted in Figure 4B, Δ*arrS* exhibited the highest abundance in the kidney (2.25 × 10^6^ CFU/g), spleen (8.27 × 10^6^ CFU/g), and liver (1.11 × 10^7^ CFU/g). The abundance of Δ*arrS* in the kidney and spleen was significantly higher than that of WT and Δ*arrS* + pBBR-*arrS*. Although no statistically significant differences were observed in the liver, Δ*arrS* still demonstrated a comparable increasing trend.

The relative levels of immune factors in serum were illustrated in Figure 4C. Infection with *A. veronii* triggered immune responses in mice that were mediated by TNF-α and IL-1β. Compared to WT and Δ*arrS* + pBBR-*arrS*, Δ*arrS* specifically induced elevated levels of TNF-α and IFN-γ. This finding corroborated the increased pathogenicity of Δ*arrS*, and suggested that TNF-α and IFN-γ might be involved in the mechanism by which Δ*arrS* enhances its pathogenicity.

The HE-stained sections of infected mice were presented in Figure 4D. Following challenge with WT, Δ*arrS*, or Δ*arrS* + pBBR-*arrS*, lesions were observed in the spleen and kidney, while no significant changes were noted in the liver. In the spleen, necrosis and disintegration were observed in the marginal zone of white pulp, accompanied by an expansion of the red pulp and an increase in blood cells and macrophages. In the kidney, the renal tubular epithelial cells in the Δ*arrS* + pBBR-*arrS* group exhibited vacuolar degeneration; while in the WT and Δ*arrS* groups, renal tubular atrophy, significant inflammatory cell infiltration in the renal interstitium, and damage to epithelial cells in the renal tubules were observed. Within the Δ*arrS* group, a larger necrotic zone was observed at the marginal zone of white pulp in spleens, accompanied by a significant expansion of red pulp; concurrently, increased bubble formation was noted due to the necrosis of glomerular epithelial cells, along with heightened infiltration of inflammatory cells into the renal interstitium, and irregularities observed in renal tubules resulting from severe wrinkling. In comparison to WT and Δ*arrS* + pBBR-*arrS*, knockout of *arrS* enhanced the pathogenicity of *A. veronii* toward the spleen and kidney of mice.

### ArrS/ArrR modulates the transcription of genes associated with virulence

**Figure 5:**
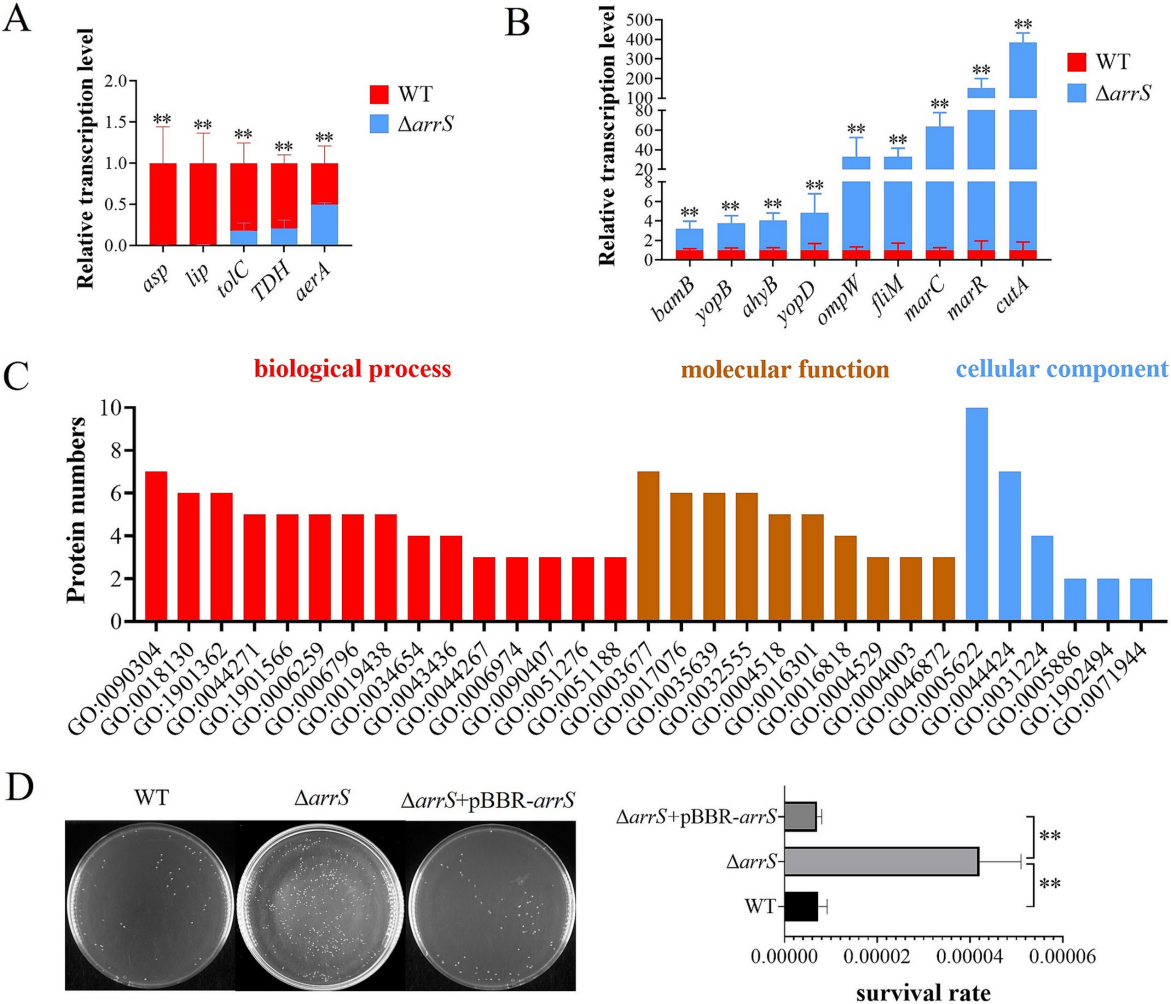
Transcriptional and posttranscriptional effects of *arrS* knockout in *A. veronii*. **(A)** RT-qPCR analysis of virulence associated genes that were down-regulated and **(B)** up-regulated. **(C)** Gene ontology annotation. The histogram depicted the differential expressed protein numbers enriched in biological process (level 5), molecular function (level 5), and cellular component term (level 3). **(D)** Verification of UV radiation tolerance. The survival rate was determined by CFU counting. Experiments were conducted with three replicates in panels **(A,B)**, and five replicates in panel **(D)**. *p*-values were obtained from t-tests and the error bars indicate the SD. **denoted extremely significant difference (*P*<0.01).

A total of twenty virulence-related genes of *A. veronii*, as previously reported, were analyzed by RT-PCR (Liu et al., 2017). Figures 5A,B illustrated the down-regulated and up-regulated genes in Δ*arrS*. The identified down-regulated genes are orthologous to the serine protease gene *asp*, the lipase gene *lip*, the outer membrane protein gene *tolC*, the thermostable direct hemolysin gene *TDH*, and the aerolysin gene *aerA*, respectively. Conversely, the up-regulated genes identified are orthologous to the outer membrane protein assembly factor gene *bamB*, the elastase gene *ahyB*, the type III secretion-related translocators genes *yopB* and *yopD*, the outer membrane protein gene *ompW*, the flagellar motor switch protein gene *fliM*, the stress protective protein gene *marC*, the multidrug resistance regulatory protein gene *marR*, and the bivalent cation tolerance protein gene *cutA*, respectively. Most of them can influence the host’s inflammatory response directly or indirectly, theoretically enhancing the immunogenicity of *A. veronii* and inducing severer autoimmune responses in host organisms.

### RecB is a target protein of the ArrS/ArrR

The fluctuation of intracellular c-di-GMP concentrations induced by ArrS/ArrR might influence the protein expression of *A. veronii*. In order to identify the target proteins of ArrS/ArrR, a proteome analysis was conducted to compare *A. veronii* WT and Δ*arrS* in the stationary phase. A total of 2,614 proteins were discovered. Among them, eight differentially expressed proteins were identified, with five up-regulated and three down-regulated in Δ*arrS*. The exonuclease RecB exhibited the most affected expression in Δ*arrS*, with a 13.8-fold increase compared to WT. Furthermore, 30 proteins were exclusively detected in Δ*arrS*, while 14 proteins were found only in WT (including ArrS). Detailed information regarding these differentially expressed proteins was listed in Supplementary Table S3. Subsequently, gene ontology annotation was performed using Blast2GO (v5.2.5), as shown in Figure 5C and Supplementary Table S4. A total of 32 proteins were annotated, and the nucleic acid metabolic process (GO:0090304) and DNA metabolic process (GO:0006259) were the most affected biological processes in *A. veronii*, involving 7 and 5 proteins, respectively. Notably, both processes included RecB.

RecB is one of the three subunits comprising exonuclease V and plays a crucial role in DNA repair. Consequently, the upregulation of RecB significantly will enhance the tolerance against UV irradiation (Prada Medina et al., 2016). Thus, we compared the UV radiation tolerance among WT, Δ*arrS*, and Δ*arrS* + pBBR-*arrS*, as presented in Figure 5D. Following 1 min irradiation, the survival rate of Δ*arrS* was found to be 0.0042%, which was approximately 5.84 times higher than that of WT and 5.97 times higher than that of Δ*arrS* + pBBR-*arrS*. This finding was consistent with the proteomic data, confirming that RecB was up-regulated in Δ*arrS* and served as a target protein of ArrR/ArrR. Thus, ArrS/ArrR might modulate the viability of *A. veronii* through RecB regulation.

## Discussion

In this study, we elucidated a TCS comprising ArrS and ArrR and investigated its function in *A. veronii*, while identified ArgR as the transcription factor. Notably, the sensor domain of ArrS featured two PBP2 domains. Previous studies have identified temperature, nicotinate, Mg^2+^, SO4^2−^, and histidine as the potential signaling molecules for PBP2 domains (Xu et al., 2017; Lesne et al., 2018). However, in this study, we were unable to validate the specific signaling molecules recognized by ArrS. Nevertheless, during the UV radiation experiments, we noticed that WT and Δ*arrS* strains were more likely to exhibit their differences in survival rates when they were undergone prolonged culture and reached high bacterial densities. Therefore, in subsequent experiments, we slightly extended the culture time of *A. veronii* to increase the likelihood of triggering the function of ArrS/ArrR. For instance, the culture time was set at 18 h for proteomic sample preparation, while it was extended to 21 h for RT-qPCR. This may account for the discrepancy between our proteomic data and RT-qPCR results.

In ArrRm, we replaced the aspartic acid residue that serves as the phosphorylation site of the REC domain with alanine. This approach was commonly used to simulate the dephosphorylated states of SK and RR. Following these mutations, the resulting simulated dephosphorylated conformation of ArrRm might be more conducive to its PDE activity compared to ArrR, leading to a slightly higher PDE activity than that of ArrR (Figure 1C). Although the differences were significant, given that the PDE activity difference between ArrRm and ArrR was less than 2-fold, we suspected that there might not be a substantial difference in their PDE activities; rather, the variation observed in Figure 1C could be attributed to fluctuations in protein expression and purification processes. For example, a fraction of ArrR might undergo phosphorylation during *in vivo* expression, leading to a reduction in the overall enzymatic activity, whereas ArrRm was less impacted due to its mutation in the phosphorylation site. Additionally, random fluctuations in PDE activity might occur during the processes of protein purification and recovery. Regardless of the existence of differences in PDE activity between ArrR and ArrRm, our conclusion that the PDE activity of ArrR was inhibited by phosphorylation remains unequivocal.

ArrS can inhibit ArrR’s PDE activity by phosphorylating ArrR, theoretically leading to a lower intracellular c-di-GMP concentration in Δ*arrS* compared to the WT. However, quantitation of intracellular c-di-GMP and observations related to biofilm and motility both indicated a higher intracellular c-di-GMP concentration in Δ*arrR*. Therefore, what is causing the increase in intracellular c-di-GMP concentration within Δ*arrR*? We suspect that ArrS may possess phosphatase activity and catalyze the dephosphorylation of ArrR. This conjecture is inspired by the behavior of BvgS: when stimulated at 37°C, BvgS demonstrates kinase activity, leading to the phosphorylation of BvgA; upon binding to nicotinate, BvgS switches to phosphatase activity and facilitates the dephosphorylation of BvgA (Dupre et al., 2015b). Given that ArrS shares similar domains with BvgS, ArrS might also have the potential to become a phosphatase like BvgS does. As previously mentioned, Figure 2F indicated ArrS and ArrR should achieve their functions in a common mechanism. This speculation further aligns well with this condition. However, we cannot rule out the possibility that alternative mechanisms contribute to elevated intracellular c-di-GMP levels in Δ*arrS*. For instance, according to the proteomic data, A0A160EYR7 and the arginine carboxylate lyase ArgH were found to be upregulated in Δ*arrS*. A0A160EYR7 has a GGDEF domain at C-terminal and might possess DGC activity, while deletion of ArgH would result in decreased c-di-GMP concentration (Barrientos-Moreno et al., 2020). Both of them possibly contribute to the elevated intracellular c-di-GMP levels in Δ*arrS*.

In this study, we have demonstrated that ArrS/ArrR can regulate the intracellular concentration of c-di-GMP *in A. veronii*, whereas the second messenger c-di-GMP is known to be closely associated with bacterial virulence. For instance, in *A. hydrophila*, c-di-GMP regulates the transcription levels of genes such as *asp*, *lip*, *aerA*, and *ahyB* through the quorum sensing systems AI-1 and AI-2 (Kozlova et al., 2011; Rama Devi et al., 2016). Similarly, in the Δ*arrS*, the transcription of these genes was affected as well. Furthermore, the down-regulation of *asp* transcription in Δ*arrS* due to increased c-di-GMP levels aligns with previous findings in *Vibrio alginolyticus* (Sheng et al., 2013). These transcriptional changes observed in Δ*arrS* reflect the regulatory function of ArrS/ArrR on c-di-GMP, further reconfirming the close relationship between intracellular c-di-GMP concentration and the transcriptional regulation of bacterial virulence.

Previous research has established that inflammation and autoimmunity in mammals can lead to tissue damage (Pastorelli et al., 2013; Christen, 2019). Notably, encoded proteins from genes up-regulated in Δ*arrS* (Figure 5B) primarily pertain to the inflammatory response: BamB has the capacity to induce the expression of IL-6 in host cells (Hsieh et al., 2016); OmpW can provoke pro-inflammatory cytokines such as TNF-α, IFN-γ, and IL-1β in carp (Zhang et al., 2019); YopB and YopD, crucial effectors of the type III secretion system, are substantial proinflammatory factors (Montagner et al., 2011; Zwack et al., 2015); FliM is the constituent proteins of the flagella C-ring, its mutations can shorten the hook length and reduce the number of assembled flagella, leading to a lower immunogenicity (Konishi et al., 2009; Rieber et al., 2013); MarR inhibits the transcription of ATP-binding cassette transporters MarC, MarA, and MarB, inducing inflammatory responses in mouse macrophages (Hao et al., 2014; Deng et al., 2020). Moreover, Δ*arrS* infection resulted in an upregulation of TNF-α and IFN-γ in mice. Both of them are pivotal players in the inflammatory response and autoimmune diseases (Jang et al., 2021; Liu et al., 2022; Zhang et al., 2022), exerting disruptive effects on epithelial tissue barrier function by inhibiting the synthesis of tight junction proteins and cadherins (Rochfort and Cummins, 2015; Ng et al., 2018; Rahman et al., 2018). Hence, we proposed that the heightened virulence of Δ*arrS* might be attributed to its enhanced immunogenicity. This heightened immunogenicity could prime phagocytosis and immune factor production, intensifying the inflammatory response and autoimmunity in the host. This hypothesis aligned with the immune factors observed in serum (Figure 4C) and was supported by findings in *A. hydrophila* (Yeremeev et al., 2015; Andersen et al., 2017; Kong et al., 2017; Bonhomme et al., 2020). Furthermore, the enhanced biofilm formation and RecB expression might contribute to the survival of Δ*arrS* within host cells, facilitating its ability to translocate via host phagocytes as carriers. Thus, we propose that ArrS/ArrR might induce a transition in *A. veronii* from an exotoxin synthesis-dominant state to a more viable and immunogenic state.

ArgR is one of the most important transcription factors in microorganisms. It not only participates in the metabolism and transport of arginine (Caldara et al., 2006), but also regulates multiple metabolic pathways in bacteria, such as glutamate synthesis, pyrimidine biosynthesis, nitrogen metabolism and nitrate utilization (Paul et al., 2007; Larsen et al., 2008; Botas et al., 2018). Furthermore, ArgR plays a critical role in regulating bacterial virulence (Mahdi et al., 2014; Dorman et al., 2018), as well as the c-di-GMP metabolism. In bacteria, ArgR has been demonstrated to regulate intracellular c-di-GMP levels through multiple distinct mechanisms. In *Pseudomonas putida*, the regulatory mechanism of ArgR is closely related to arginine metabolism. It was reported that sufficient L-arginine will promote the synthesis of c-di-GMP, whereas ArgR can reduce the intracellular c-di-GMP levels by inhibiting L-arginine production (Barrientos-Moreno et al., 2020); Moreover, the elevated c-di-GMP levels will promote the expression of ArgR in the presence of FleQ, acting as a feedback regulator (Barrientos-Moreno et al., 2022). In *A. veronii*, recent study has reported that ArgR can decrease intracellular c-di-GMP levels by repressing the transcription of multiple DGC genes (Wang et al., 2024). This regulatory mechanism primarily emphasizes ArgR’s function as a transcription factor. In this study, we further elucidated its role as a transcription factor in c-di-GMP metabolism. Since ArgR was identified as a transcription repressor of the *arrSR* operon, it could prevent the degradation of c-di-GMP by inhibiting *arrSR* regulon. In conclusion, ArgR has been demonstrated to play a dual role in both positively and negatively regulating c-di-GMP metabolism. Consequently, L-arginine, ArgR, and c-di-GMP likely constitute a complex self-regulating feedback loop that enables precise arginine-dependent regulation of c-di-GMP.

**Figure 6:**
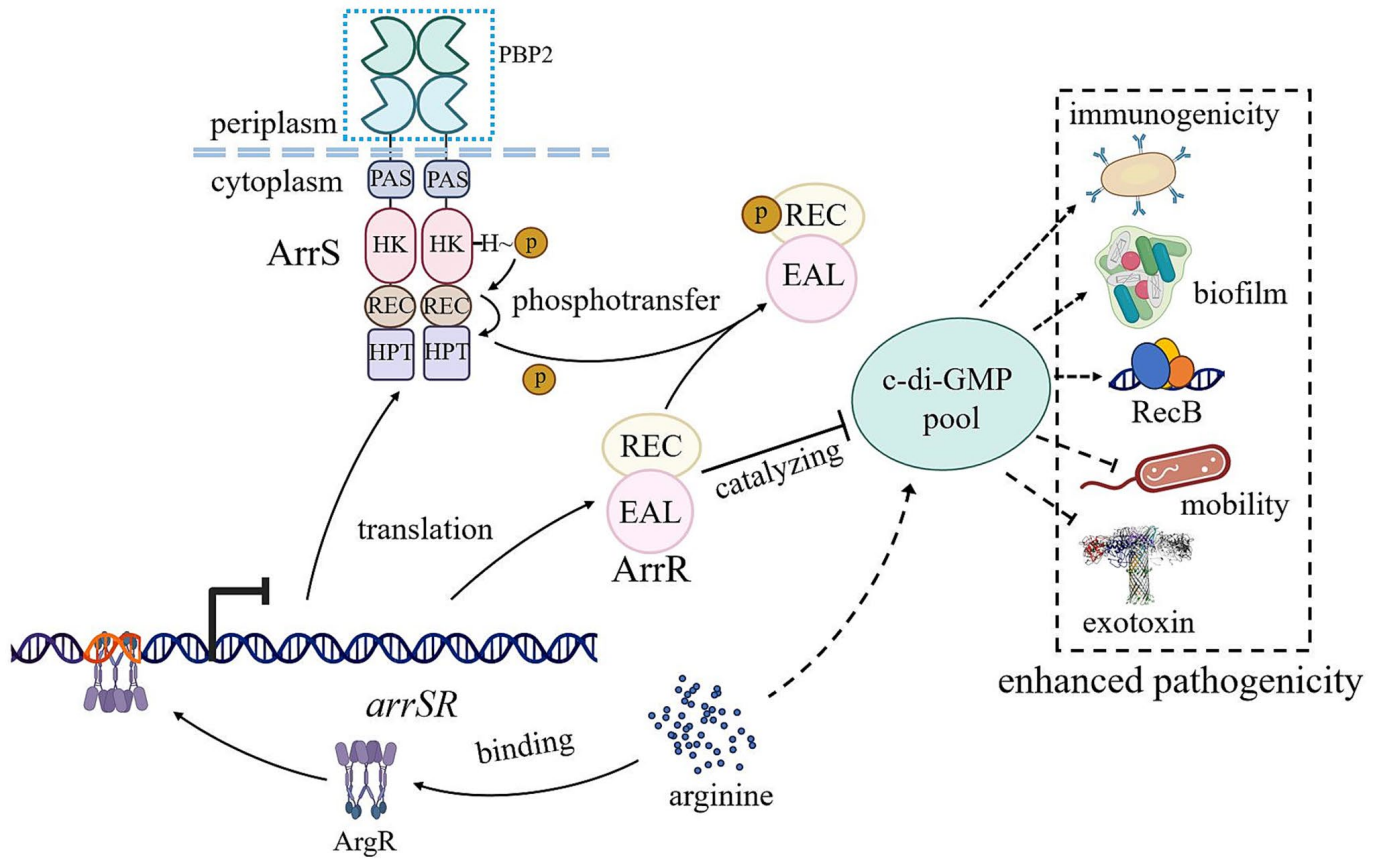
Schematic representation of ArrS/ArrR regulation and function. The transcription of *arrSR* is regulated by the arginine-dependent transcription factor ArgR. Following expression, ArrS modulates the phosphorylation status of ArrR to finely tune its PDE activity, controlling the intracellular concentration of c-di-GMP. The phosphorylated ArrR leads to increased c-di-GMP levels in *vivo*, promoting biofilm formation, RecB expression, and immunogenicity in *A. veronii* while impairing motility and exotoxin synthesis, ultimately enhancing pathogenicity against mice. Arrows and T-lines represented positive and negative regulation, respectively. Solid lines indicated direct effects, while dotted lines denoted indirect effects or effects with undefined mechanisms.

In this study, we identified a novel TCS comprising ArrS and ArrR in *A. veronii*, assessed its impact on intracellular c-di-GMP levels, and found that ArgR served as its transcriptional repressor. Furthermore, we conducted a preliminary investigation into its virulence regulatory function. In conclusion, we presented a schematic diagram (Figure 6) illustrating the regulatory relationships involving ArrS/ArrR. When intracellular arginine levels are low, the inhibitory effect of ArgR on P*_arrS_* is relieved, leading to the initiation of *arrSR* transcription. At this point, *A. veronii* can modulate intracellular c-di-GMP levels via the ArrS/ArrR system, thereby influencing physiological processes such as biofilm formation, motility, and virulence.

## Data Availability

ChIP-seq data reported in this paper have been deposited at the Gene Expression Omnibus (GEO) Database, accession number GSE277129. The mass spectrometry proteomics data have been deposited to the ProteomeXchange Consortium via the iProX partner repository with the dataset identifier PXD059577. The data that support the findings of this study are available from the authors upon reasonable request.
