# Supplementary material for: A novel two-component system contributing the catabolism of c-di-GMP influences virulence in Aeromonas veronii
Source: Front Microbiol. 2025 Jan 30;16:1527317. doi: 10.3389/fmicb.2025.1527317 (PMC11841396; doi:10.3389/fmicb.2025.1527317)
Supplement: Supplementary file 1 [file Data_Sheet_1.docx]

**Supplementary information**

**Table S1 strains and plasmids**

| **Name** | **Description** | **Reference** |
| --- | --- | --- |
| ***Aeromonas veronii*** | | |
| WT | *Aeromonas veronii* C4 | Lab stock |
| Δ*arrS* | C4 derivative with a deletion mutant in *arrS* gene | This study |
| Δ*arrS*+pBBR-*arrS* | Δ*arrS* derivative complemented with pBBR-*arrS* | This study |
| Δ*arrR* | C4 derivative with a deletion mutant in *arrR* gene | This study |
| Δ*arrR*+pBBR-*arrR* | Δ*arrR* derivative complemented with pBBR-*arrR* | This study |
| Δ*arrS*Δ*arrR* | C4 derivative with both *arrS* and *arrR* knocked out | This study |
| Δ*argR* | C4 with a deletion mutant in the *argR* gene | Lab stock |
| Δ*argR*+pBBR-*argR* | Δ*argR* derivative complemented with pBBR-*argR* | This study |
| ***Escherichia coli*** | | |
| WM3064 | *thrB1004 pro thi rpsL hsdS lacZ*Δ*M15 RP4-1360* Δ(*araBAD*)*567* Δ*dapA1341*::[*erm pir*] | Lab stock |
| DH5α | *endA hsdR17 supE44 thi*-*1 recA1 gyrA relA1*Δ(*lacZYA-argF*)*U169 deoR* (*φ80dlac*Δ(*lacZ*)*M15*) | Lab stock |
| BL21(DE3) | *F*–*ompT hsdS_B_*(*r_B_*^-^*m_B_*^-^) *gal dcm met* (*DE3*) *endA1 gyrA96*(*nalR*) *thi-1 recA1 relA1 lac glnV44 e14*- | Lab stock |
| XL1-Blue MRF' | Δ(*mcrCB*-*hsdSMR*-*mrr*)*171 recB recJ sbcC umuC*::*Tn5 uvrC F'*[ ::*Tn10 proAB*+ *lacIq* Δ(*lacZ*)*M15 Amy CmR*] | Lab stock |
| **Plasmid** | | |
| pRE112 | Cm^R^; Bacterial allelic exchange vector with *sacB* | Lab stock |
| pRE112-*arrS* | pRE112 with two homologous fragments of *arrS* | This study |
| pRE112-*arrR* | pRE112 with two homologous fragments of *arrR* | This study |
| pBBR1MCS-2 | Km^R^; Mobilisable shuttle and expression vector | Lab stock |
| pBBR*-arrS* | pBBR1MCS-2 derivative constitutive expressing ArrS | This study |
| pBBR*-arrR* | pBBR1MCS-2 derivative constitutive expressing ArrR | This study |
| pBBR*-arrR*m | pBBR1MCS-2 derivative constitutive expressing ArrR with mutated phosphorylation sites. | This study |
| pBBR*-arrR*^aal^ | pBBR1MCS-2 derivative constitutive expressing ArrR with mutated EAL domain. | This study |
| pBBR*-argR* | pBBR1MCS-2 derivative constitutive expressing ArgR | This study |
| pBBR-*dgcH* | pBBR1MCS-2 derivative constitutive expressing DgcH from *P. aeruginosa* | This study |
| pBBR*-*P*_cdrA_*-GFP | pBBR1-MSC2 with a GFP expression system controlled by *cdrA* promoter in *P. aeruginosa* PAO1 | This study |
| pET28a | Km^R^; Expression vector with *lacZ* operon, T7 promoter and His-Tag | Lab stock |
| pET28a-ArrS | pET28a derivative expressing ArrS^820-1414^ protein | This study |
| pET28a-ArrR | pET28a derivative expressing ArrR protein | This study |
| pET28a-ArrRm | pET28a derivative expressing mutanted ArrR protein | This study |
| pET28a-ArgR | pET28a derivative expressing ArgR protein | Lab stock |
| pBT | Cm^R^; Bait plasmid carrying λcI protein | Lab stock |
| pBT-LGF2 | pBT derivative as positive control in two-Hybrid System | Lab stock |
| pBT-REC | pBT derivative with the REC domain of ArrR fused to λcI | This study |
| pBT-ArgR | pBT derivative expressing ArgR protein | Lab stock |
| pTRG | Tet^R^; Target plasmid carrying the N-terminal domain of RNAPα | Lab stock |
| pTRG-Gal11^p^ | pTRG derivative as positive control in two-Hybrid System | Lab stock |
| pTRG-HPT | pTRG derivative with the HPT domain of ArrS fused to RNAPα | This study |
| pTRG-ArgR | pTRG derivative with ArgR fused to RNAPα | Lab stock |
| pTRG-SmpB | pTRG derivative with SmpB fused to RNAPα |  |
| pDH114 | Km^R^; Expression vector with a GFP expression system controlled by *arrS* promoter | Lab stock |
| pDH114m | pDH114 derivative with site-mutant in ARG-BOX | This study |
| pBXcmT | pBT derivative one-hybrid screening report vector | Lab stock |
| pBXcmT-P*_arrS_* | pBXcmT derivative carrying *arrS* promoter | This study |
| pBXcmT-P*_arrS_*m | pBXcmT-P*_arrS_* with point mutation in ARG-BOX | This study |

**Table S2 Primers**

| **Primer** | **Sequence (5’– 3’)** |
| --- | --- |
| *arrS-*F0 | AGGTGGCCCTTTTTATTTGTCC |
| *arrS-*R0 | GGCAATAACGACCCGATCGAC |
| *arrS*arm1-F | ACCTTAGCCCAGGTACCTGGTTGCCATCCCGGTACTAGCC |
| *arrS*arm1-R | ATCACATCCCAATTAAATGGAAACCTTGAAGCGCAGGGTC |
| *arrS*arm2-F | ATCACATCCCATTTAATTGGCGGCACGTCTCTCTCCATC |
| *arrS*arm2-R | CCTCACCACCAGAGCTCTGGCCGGAATAAAATCGATCTGC |
| *arrR-*F0 | ATGGGCTCGGAGAACTGGTG |
| *arrR-*R0 | ATGGTGACTCAAGGCATGCG |
| *arrR*arm1-F | GCCCGATCCCAAGCTTCTTCTAGATGATCACCGACTGCCACAT |
| *arrR*arm1-R | AAAGGGTTGGTCGCAGGAGAATGATGACCAACTCGTCTGC |
| *arrR*arm2-F | TCTCCTGCGACCAACCCTTT |
| *arrR*arm2-R | CTGCATGAATTCCCGGGAGAGCTCCATGGCGTCATAACTCGCTT |
| pRE112-F | ACATAGCCCCACTGTTCGT |
| pRE112-R | TTTTCGTCTCAGCCAATCC |
| *arrS*-F | GAATTCCCGGGGATCCTCGAGTATGCTCCGCCAATGTGATCG |
| *arrS*-R | GAATTCCCGGGGATCCTCGAGTATGCTCCGCCAATGTGATCG |
| *A. veronii*-F | ATGGTCGCAGAGCTTGTC |
| *A. veronii*-R | CAGCACAATAGAACACCAGAC |
| pBBR-F | GGCACCCCAGGCTTTACACT |
| pBBR-R | GATGTGCTGCAAGGCGATTAAG |
| pBBR*arrS*-F | AGCCTCGAGAAAGCGCCAATCCTGATCC |
| pBBR*arrS*-R | AACTTGGTACCTCTGCCGTTCTACTCATCG |
| pBBRP*_arrS_*-F | TAAAGGGAACAAAAGCTGGGTACCTTATCATCAGGTTGCCATCC |
| pBBRP*_arrS_*-R | CTCGTCTGCCGTTCTACTCATCAGAAGGAATCCAACAGAAT |
| pBBR*arrR*-F | ATGAGTAGAACGGCAGACGAG |
| pBBR*arrR*-R | CTTATCGATACCGTCGACCTCGAGGATCAGCCGGATATCAGGGTC |
| P*_cdrA_*GFP-F | GAATTCCTGCAGCCCGGGGGATCCAGGCATGGTCGAGGAAAACG |
| P*_cdrA_*GFP-R | CACCGCGGTGGCGGCCGCTCTAGATTACTTGTACAGCTCGTCCAT |
| DgcH-F | AAGCTGGGTACCGATGAGTCGCGACGACGTCCA |
| DgcH-R | ACTAGTGGATCCGTCGGAATGTGCGCTGGT |
| P*_arrS_*-F | ACCACGCAGGCTTTATCATC |
| P*_arrS_*-R | ATCACATTGGCGGAGCAT |
| P*_cdrA_*-F | CAGTCGGGAAACCTGTCGTGGGTACCCTGCCTAGCCTAGCAAACC |
| P*_cdrA_*-R | CTGGCTATCCGGACGGACC |
| GFP-F | GGTCCGTCCGGATAGCCAGATGGTGAGCAAGGGCGAGGA |
| GFP-R | TAACCAATAGGCCGACTGCGGGATCCTTACTTGTACAGCTCGTCCAT |
| AGRm-F | ATTGATGTTGGCCGCTGCTATTTAATTCACTTACAATAT |
| AGRm-R | ATATTGTAAGTGAATTAAATAGCAGCGGCCAACATCAAT |
| ArgR-F0 | TTGCGAGAAGTCCTTCACTC |
| ArgR-R0 | CAAGCAAGATGAAACGGGAG |
| pBBR*argR*-F | TCCTTGGTACCGGTGGTCAAAACCCTCAGTCGT |
| pBBR*argR*-R | CCTAAGGATCCCAGGTCGCCTCAGTGTATTT |
| ArrRm-F | TCGACATCCTGTTCTGCGCTCTGCGGATGCCCGG |
| ArrRm-R | CCGGGCATCCGCAGAGCGCAGAACAGGATGTCGA |
| ArrRm2-F | TGGTCATCATGGTGGTCGCGGCTCACGGGTTTCAACGCAA |
| ArrRm2-R | TTGCGTTGAAACCCGTGAGCCGCGACCACCATGATGACCA |
| AAL-F | GCCAGTGGGTGGGGATGGcGGCGCTGGCCCGCTGGCA |
| AAL-R | TGCCAGCGGGCCAGCGCCgCCATCCCCACCCACTGGC |
| pTRGHPT-F | AAACCAGAGGCGGCCGGATCCTCCGCGACTACGATCACAGAG |
| pTRGHPT-R | TTAATTAATTAATTACTCGAGCTACTCATCGAGCCACAATCCC |
| pBTREC-F | TGGCGCGGCCGCATCGAATTCCATGAGTAGAACGGCAGACGAGTT |
| pBTREC-R | AATTAATTAACTCGAGGATCCTGCCCAGGAGTCGATCAACTG |
| Riboswich-F | CCGCTCTAGAACTAGTGGTACCGGGCTCATTCTCACATTT |
| Riboswich-R | TCCTCGCCCTTGCTCACCATGCTGTTAGTCTCGGAGTAT |
| RSGFP-F | ATACTCCGAGACTAACAGCATGGTGAGCAAGGGCGAGGA |
| RSGFP-R | AAGGGAACAAAAGCTGGGTACCTTACTTGTACAGCTCGTC |
| pETArrS-F | TTGTGGATCCGCTCGCAGCCGTTTTCTGG |
| pETArrS-R | AAGCTCTCGAGCTCATCGAGCCACAATCCCAT |
| pETArrR-F | AACAGGATCCATGAGTAGAACGGCAGACGAG |
| pETArrR-R | ACATCCTCGAGGGATATCAGGGTCGGCAGTC |
| control-F | TTGTCGATCGCTGCTATGCC |
| control-R | TTGCTATCTGGAGCAGGGTT |
| RT*arrS*-F | GCACTAGTCAGCCTGGTGAGC |
| RT*arrS*-R | ATCAGGTCCGCCACATAACCG |
| RT*arrR*-F | ACAAGTCGCTACAGGATTGCC |
| RT*arrR*-R | AACGAGCTTTCGGTCACCTC |
| RT*recB*-F | CAAGATAGCTGACTGGGCACAGG |
| RT*recB*-R | CGTCGAGGTCTTTGAGCAGGT |
| RT*asp*-F | GAACACCTCCAACTACAA |
| RT*asp*-R | GTATAGAAGTCGGTCTTGATA |
| RT*tolC*-F | CCTCTATCGTCGCAGTAAC |
| RT*tolC*-R | GGTATCCATCGCCTTGAG |
| RT*hyl*-F | CCTCTATCGTCGCAGTAAC |
| RT*hyl*-R | GGTATCCATCGCCTTGAG |
| RT*lip*-F | GCACCAGTCACGACAATG |
| RT*ahyB*-F | GTGTATCAGGTCTCCTTCATC |
| RT*ahyB*-R | GGCATCCTTGTGGTTGAG |
| RT*ompW*-F | CAGGTCAGGTCGGTATCG |
| RT*ompW*-R | TTAGAAGCGGTAGCCAACA |
| RT*bamB*-F | TGTTCAGTTCCGAAGAGG |
| RT*bamB*-R | AGTAGAAGTCACCGATGC |
| RT*yopB*-F | ACTGGCGGTAAGCAATAG |
| RT*yopB*-R | CTTGGTCTGGGTCTTCTC |
| RT*yopD*-F | GCACAGGTTGATGAGATG |
| RT*yopD*-R | GGATTGTTGCTGCTTGATA |
| RT*fliM*-F | CGACTGGTCTTCATCCTG |
| RT*fliM*-R | GGCATCCTTGTAATCCTCA |
| RT*cutA*-F | TCATCAAATCCCGTCAATC |
| RT*cutA*-R | CGAGATAGGCAGGTAGTC |
| RT*marR*-F | ATGACGGATCTGGAAAGG |
| RT*marR*-R | TTCTGGCTGACATGGTTC |
| RT*marC*-F | CTACGCAGGTGTGGTAAC |
| RT*marC*-R | ACTTCCTTGATGCCATTGA |

**Table S3 differentially expressed proteins**

| **Protein ID** | **Name** | **Description** | **Detected in**  **WT or Δ*arrS*** | **Fold change**  **(Δ/WT)** | **P-value** |
| --- | --- | --- | --- | --- | --- |
| A0A142DZE8 | *recB* | RecBCD enzyme subunit RecB | Both | 13.8030 | 0.0085 |
| A0A160EYR7 |  | Diguanylatecyclase | Both | 2.5105 | 0.0323 |
| A0A0E2LG95 |  | Uncharacterizedprotein | Both | 2.4648 | 0.0306 |
| A0A223LYH4 | *argH* | Argininosuccinate lyase | Both | 2.1975 | 0.0433 |
| A0A0T6PMU8 | *Hcp* | Hcp1 family typeVI secretion system effector | Both | 2.0232 | 0.0005 |
| A0A160F0K3 |  | NAD(P)-dependent dehydrogenase | Both | 0.4835 | 0.0411 |
| A0A0E2LRQ8 | *cpxR* | Transcriptional regulator | Both | 0.4739 | 0.0312 |
| A0A1Q8F4G0 | *oprD* | OprD family outer membrane porin | Both | 0.4504 | 0.0327 |
| A0A0E2LDM6 | *EF-P* | Translation elongation factor P | Δ*arrS* only |  |  |
| A0A0E2LJI4 | *mobA* | Molybdenum cofactor guanylyltransferase | Δ*arrS* only |  |  |
| A0A160EXX2 | *folA* | type 3 dihydrofolate reductase | Δ*arrS* only |  |  |
| K1JPQ4 | *pdxB* | Erythronate-4-phosphate dehydrogenase | Δ*arrS* only |  |  |
| A0A0E2LEV2 | *ribF* | FMN adenylyltransferase | Δ*arrS* only |  |  |
| A0A0E2LHB9 | *araC* | Carbamate kinase | Δ*arrS* only |  |  |
| A0A0E2LTW4 | *flgN* | Flagellar biosynthesis protein | Δ*arrS* only |  |  |
| Q6XL58 | *cphA6* | Beta-lactamase | Δ*arrS* only |  |  |
| A0A0E2LBH5 | *luxR* | LuxR family transcriptional activator | Δ*arrS* only |  |  |
| A0A0E2LM90 | *cadA* | Lysine decarboxylase CadA | Δ*arrS* only |  |  |
| A0A160EWE6 | *recD* | RecBCD enzyme subunit RecD | Δ*arrS* only |  |  |
| A0A0T6QUA6 | *sirA* | tRNA 2-thiouridine synthesizing protein A | Δ*arrS* only |  |  |
| A0A1Q8EZ44 | *purH* | Bifunctional purine biosynthesis protein | Δ*arrS* only |  |  |
| A0A0E2LD32 | *nadC* | nicotinate-nucleotide pyrophosphorylase | Δ*arrS* only |  |  |
| A0A1N7AVT9 | *acuI* | Acrylyl-CoA reductase (NADPH) | Δ*arrS* only |  |  |
| K1K065 |  | MOSC domain-containing protein | Δ*arrS* only |  |  |
| A0A0E2M0I2 |  | LexA family transcriptional repressor | Δ*arrS* only |  |  |
| A0A0E2LBD7 |  | LapD/MoxY domain protein | Δ*arrS* only |  |  |
| A0A1Q8EZS6 |  | Nucleoside-diphosphate sugar epimerase | Δ*arrS* only |  |  |
| A0A0E2LGG3 |  | Integrase | Δ*arrS* only |  |  |
| A0A1Q8F666 |  | Uncharacterized protein | Δ*arrS* only |  |  |
| A0A1Q8F1M7 |  | GNAT family N-acetyltransferase | Δ*arrS* only |  |  |
| A0A142E3I7 |  | Hydrogenas | Δ*arrS* only |  |  |
| A0A165SZZ7 |  | Membraneprotein | Δ*arrS* only |  |  |
| A0A0E2LGC0 |  | Electrontransport complex subunit E | Δ*arrS* only |  |  |
| A0A0E2LER5 |  | Thiamin phosphate synthase | Δ*arrS* only |  |  |
| A0A0E2LL92 |  | membrane-anchored protein | Δ*arrS* only |  |  |
| A0A1Q8F756 |  | Uncharacterized protein | Δ*arrS* only |  |  |
| K1IW44 |  | Uncharacterized protein | Δ*arrS* only |  |  |
| A0A0E2LM44 |  | Uncharacterized protein; | Δ*arrS* only |  |  |
| A0A0E2LJT9 | *arrS* | Histidine kinase | WT only |  |  |
| A0A0E2LDB5 | *flgD* | Basal-body rod modification protein | WT only |  |  |
| A0A0E2LGK5 | *cydD* | Cysteine/glutathione ABC transporter permease | WT only |  |  |
| A0A0E2LLS7 | *phoR* | Phosphate regulon sensor protein | WT only |  |  |
| A0A1Q8EZJ1 | *gmhD* | ADP-L-glycero-D-manno-heptose-6-epimerase | WT only |  |  |
| A0A0E2LDH2 |  | Serine protease | WT only |  |  |
| A0A0E2LI00 |  | von Willebrand factor A | WT only |  |  |
| A0A0E2LS10 |  | Acyltransferase | WT only |  |  |
| A0A0E2LTY1 |  | Membrane protein | WT only |  |  |
| A0A1N6NDI8 |  | DNA repair protein | WT only |  |  |
| A0A0E2LAG5 |  | Bac_luciferase domain-containing protein | WT only |  |  |
| A0A1N6P9Z8 |  | TatD Dnase family protein | WT only |  |  |
| A0A142E572 |  | Uncharacterized protein | WT only |  |  |
| A0A1Q8F725 |  | Uncharacterized protein | WT only |  |  |
| A0A1Q8FAI4 |  | Uncharacterized protein | WT only |  |  |

Table S4 Details for GO annotation

| **Type** | **GO_ID** | **Term** | **Sequences** |
| --- | --- | --- | --- |
| Biological Process | GO:0090304 | nucleic acid metabolic process | A0A2S3XLS1, A0A0E2LLS7, A0A0E2LGG3, A0A0T6QUA6, A0A160EWE6, A0A0E2LB43, A0A1N6P9Z8 |
| Biological Process | GO:0018130 | heterocycle biosynthetic process | A0A1Q8EZ44, K1JPQ4, A0A0E2LLS7, A0A0E2LD32, A0A0E2LJI4, A0A1Q8EZJ1 |
| Biological Process | GO:1901362 | organic cyclic compound biosynthetic process | A0A1Q8EZ44, K1JPQ4, A0A0E2LLS7, A0A0E2LD32, A0A0E2LJI4, A0A1Q8EZJ1 |
| Biological Process | GO:0044271 | cellular nitrogen compound biosynthetic process | A0A1Q8EZ44, K1JPQ4, A0A0E2LLS7, A0A0E2LD32, A0A1Q8EZJ1 |
| Biological Process | GO:1901566 | organonitrogen compound biosynthetic process | A0A1Q8EZ44, K1JPQ4, A0A223LYH4, A0A0E2LD32, A0A0E2LJI4 |
| Biological Process | GO:0006259 | DNA metabolic process | A0A2S3XLS1, A0A0E2LGG3, A0A160EWE6, A0A0E2LB43, A0A1N6P9Z8 |
| Biological Process | GO:0006796 | phosphate-containing compound metabolic process | A0A1Q8EZ44, A0A0E2LJT9, A0A0E2LLS7, A0A0E2LD32, A0A0E2LJI4 |
| Biological Process | GO:0019438 | aromatic compound biosynthetic process | A0A1Q8EZ44, K1JPQ4, A0A0E2LLS7, A0A0E2LD32, A0A1Q8EZJ1 |
| Biological Process | GO:0034654 | nucleobase-containing compound biosynthetic process | A0A1Q8EZ44, A0A0E2LLS7, A0A0E2LD32, A0A1Q8EZJ1 |
| Biological Process | GO:0043436 | oxoacid metabolic process | A0A0E2LM90, A0A0E2LHB9, A0A223LYH4, A0A160EXX2 |
| Biological Process | GO:0044267 | cellular protein metabolic process | A0A0E2LJT9, A0A0E2LLS7, A0A0E2LJI4 |
| Biological Process | GO:0006974 | cellular response to DNA damage stimulus | A0A2S3XLS1, A0A160EWE6, A0A0E2LB43 |
| Biological Process | GO:0090407 | organophosphate biosynthetic process | A0A1Q8EZ44, A0A0E2LD32, A0A0E2LJI4 |
| Biological Process | GO:0051276 | chromosome organization | A0A2S3XLS1, A0A160EWE6, A0A0E2LB43 |
| Biological Process | GO:0051188 | cofactor biosynthetic process | K1JPQ4, A0A0E2LD32, A0A0E2LJI4 |
| Molecular Function | GO:0003677 | DNA binding | A0A2S3XLS1, A0A0E2LBH5, A0A0E2LGG3, A0A0E2M0I2, A0A0E2LRQ8, A0A160EWE6, A0A0E2LB43 |
| Molecular Function | GO:0017076 | purine nucleotide binding | A0A2S3XLS1, A0A0E2LLS7, A0A0E2LGK5, A0A160EWE6, A0A0E2LB43, A0A0E2LJI4 |
| Molecular Function | GO:0035639 | purine ribonucleoside triphosphate binding | A0A2S3XLS1, A0A0E2LLS7, A0A0E2LGK5, A0A160EWE6, A0A0E2LB43, A0A0E2LJI4 |
| Molecular Function | GO:0032555 | purine ribonucleotide binding | A0A2S3XLS1, A0A0E2LLS7, A0A0E2LGK5, A0A160EWE6, A0A0E2LB43, A0A0E2LJI4 |
| Molecular Function | GO:0004518 | nuclease activity | A0A2S3XLS1, A0A160EWE6, A0A0E2LB43, A0A1Q8FAI4, A0A1N6P9Z8 |
| Molecular Function | GO:0016301 | kinase activity | A0A0E2LEV2, A0A0E2LHB9, A0A0E2LJT9, A0A0E2LLS7, A0A160EXX2 |
| Molecular Function | GO:0016818 | hydrolase activity, acting on acid anhydrides, in phosphorus-containing anhydrides | A0A2S3XLS1, A0A0E2LGK5, A0A160EWE6, A0A0E2LB43 |
| Molecular Function | GO:0004529 | exodeoxyribonuclease activity | A0A2S3XLS1, A0A160EWE6, A0A0E2LB43 |
| Molecular Function | GO:0004003 | ATP-dependent DNA helicase activity | A0A2S3XLS1, A0A160EWE6, A0A0E2LB43 |
| Molecular Function | GO:0046872 | metal ion binding | A0A2S3XLS1, K1IW44, A0A0E2LJI4 |
| Cellular Component | GO:0005622 | intracellular | A0A0E2LM90, K1JPQ4, A0A0E2LJT9, A0A223LYH4, A0A0E2LLS7, A0A0T6QUA6, A0A0E2LRQ8, A0A160EWE6, A0A0E2LB43, A0A0E2LJI4 |
| Cellular Component | GO:0044424 | intracellular part | A0A0E2LM90, K1JPQ4, A0A223LYH4, A0A0T6QUA6, A0A160EWE6, A0A0E2LB43, A0A0E2LJI4 |
| Cellular Component | GO:0031224 | intrinsic component of membrane | A0A0E2LGC0, A0A0E2LJT9, A0A0E2LLS7, A0A0E2LGK5 |
| Cellular Component | GO:0005886 | plasma membrane | A0A165SZZ7, A0A0E2LGC0 |
| Cellular Component | GO:1902494 | catalytic complex | A0A160EWE6, A0A0E2LB43 |
| Cellular Component | GO:0071944 | cell periphery | A0A165SZZ7, A0A0E2LGC0 |


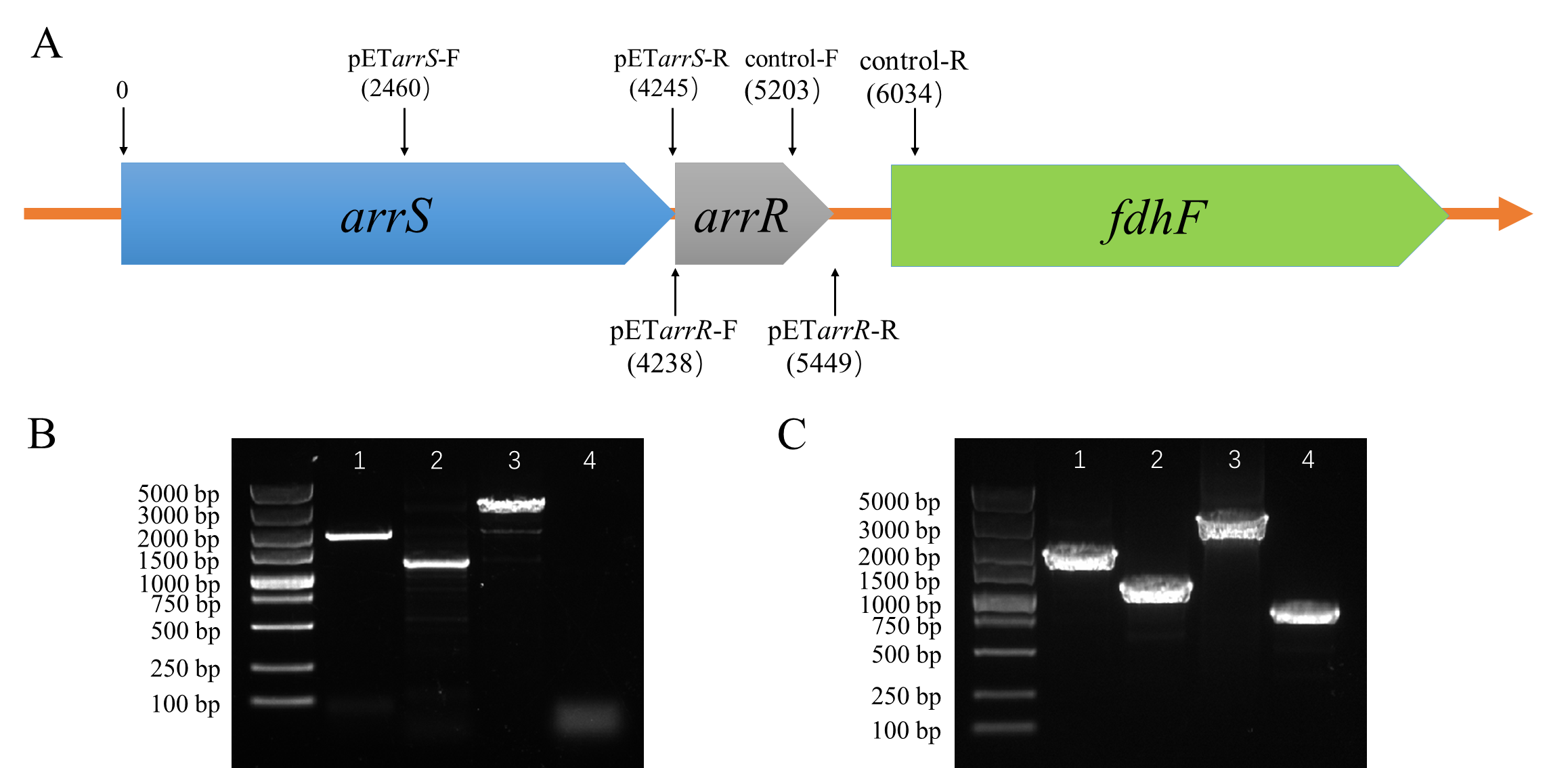


**Figure S1 Co-transcription of *arrS* and *arrR*.** The PCR products were amplified using the primers pET*arrS*-F/R, pET*arrR*-F/R, pET*arrS*-F/pET*arrR*-R and control-F/R, which were subsequently numbered 1 to 4, respectively. The primer locations were illustrated in **(A)**, while the PCR products generated from cDNA and genome were illustrated in **(B)** and **(C)**, respectively.


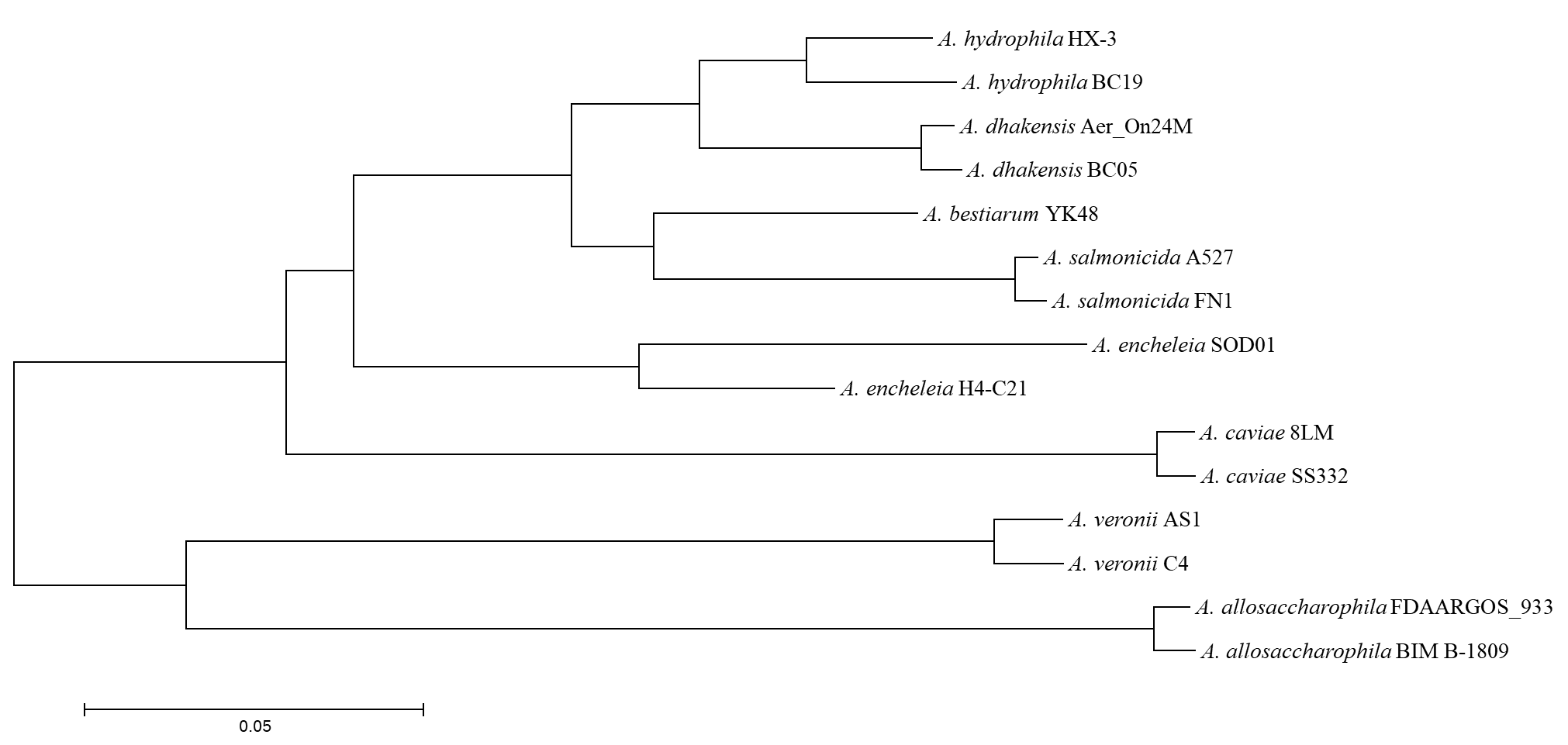


**Figure S2 Phylogenetic tree of *arrSR* in *Aeromonas*.** ArrS and ArrR were identified in 15 of the known 36 *Aeromonas*. 15 strains from 8 commonly found *Aeromonas* were selected to draw the neighbor-joining phylogenetic tree of *arrSR*, which was constructed using MEGA v7.0.26.


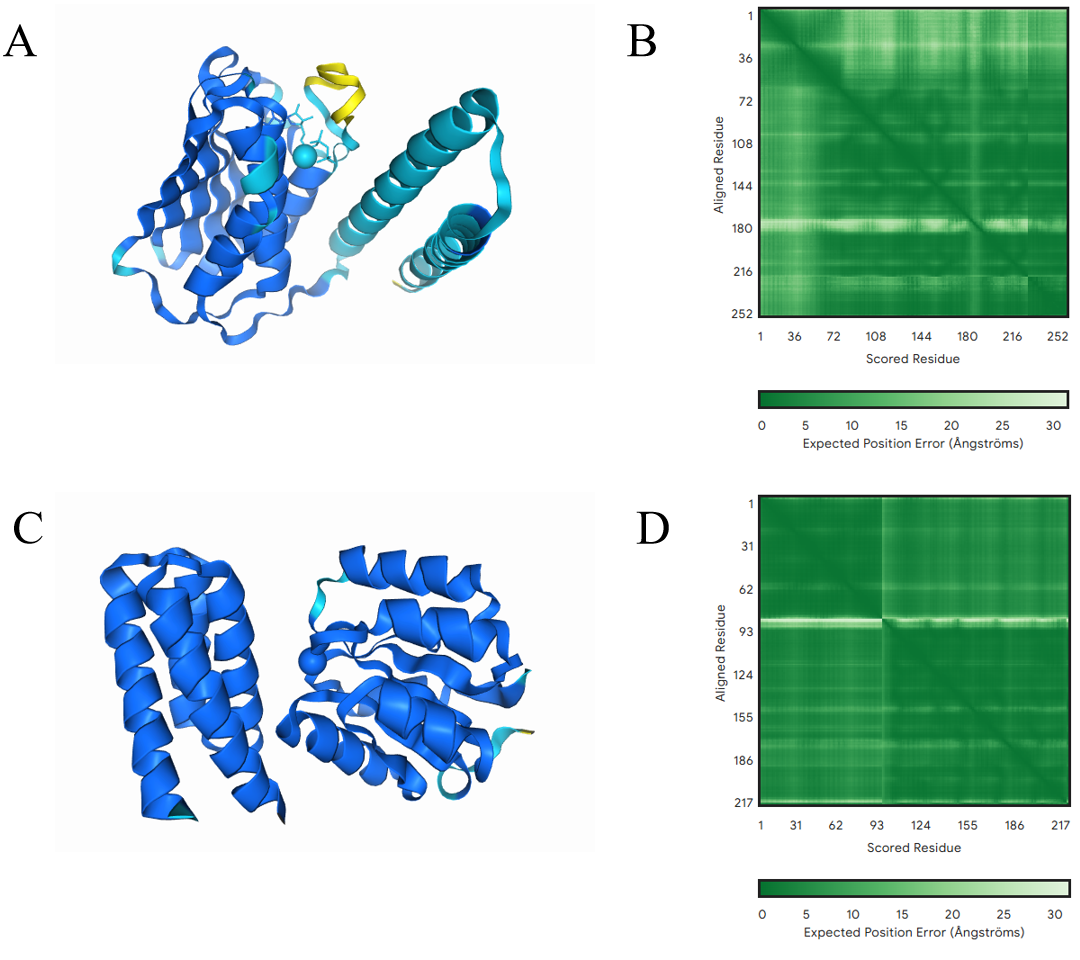


**Figure S3 Predicted models and their confidence metrics. (A)** and **(C)** show the pLDDT scores for the autophosphorylation and the interaction models, respectively. The blue regions indicate pLDDT scores greater than 90, the cyan regions indicate pLDDT scores between 70 and 90, and the yellow regions indicate pLDDT scores between 50 and 70. **(B)** and **(D)** present the corresponding PAE plots.


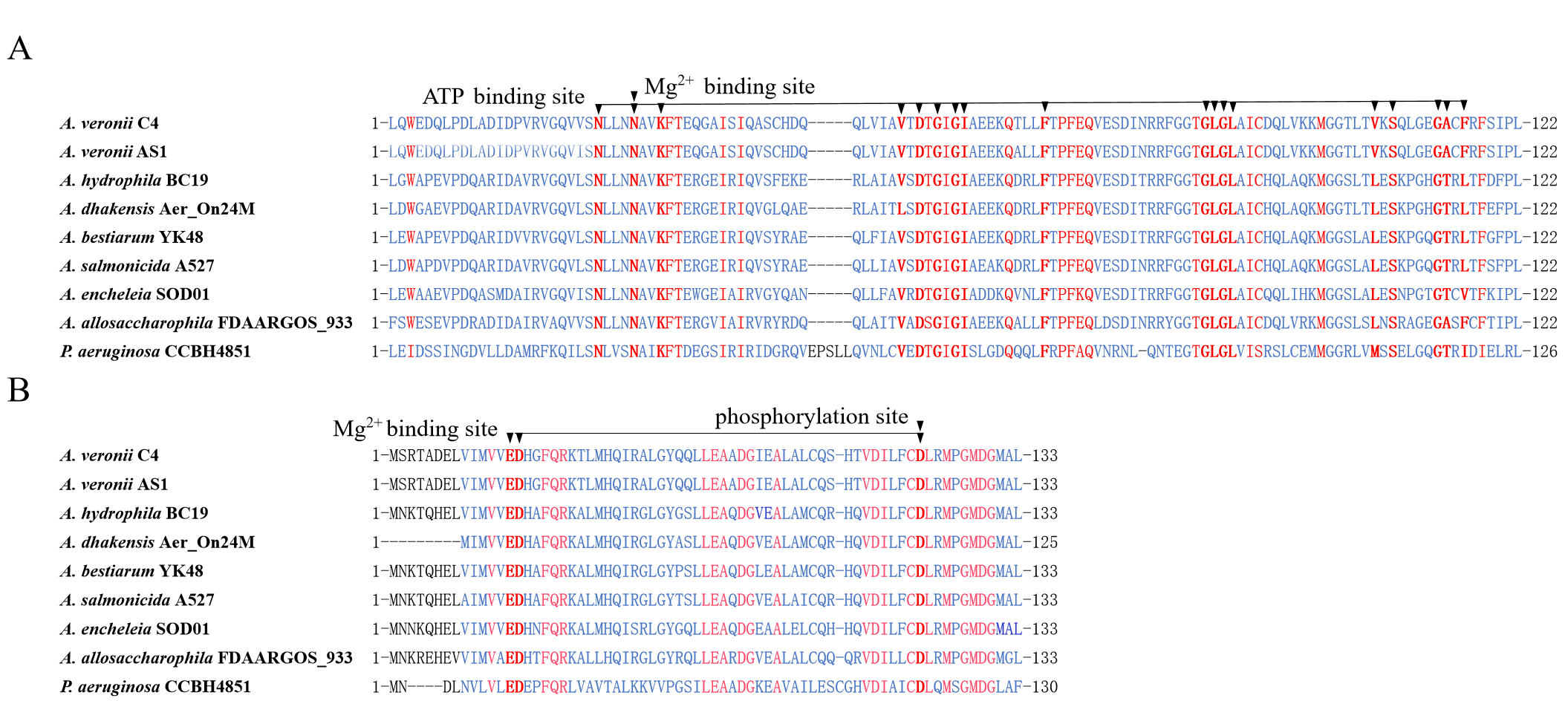


Figure S4 Conserved domain annotation via CD-search. (A) The HK domain of ArrS. (B) The REC domian of ArrR.


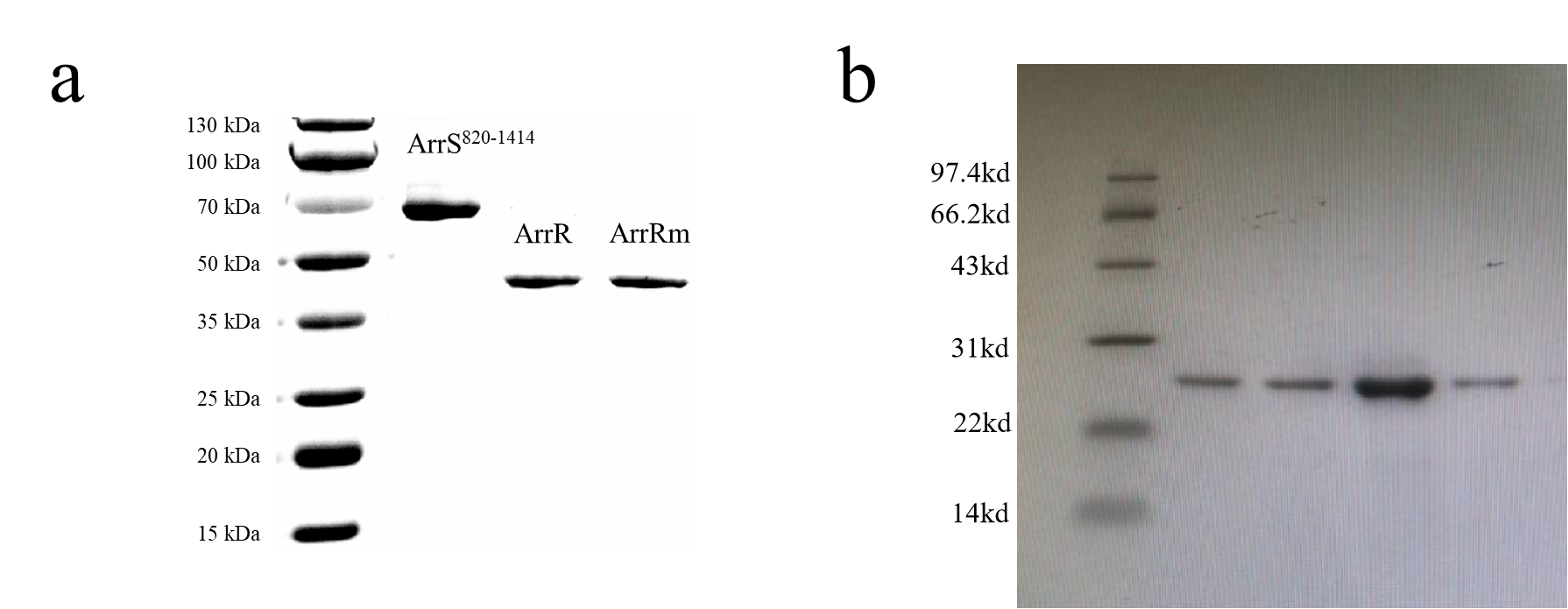


**Figure S5 Protein expression and purification. (A)** ArrS^820-1414^, ArrR and ArrRm. **(B)** ArgR.


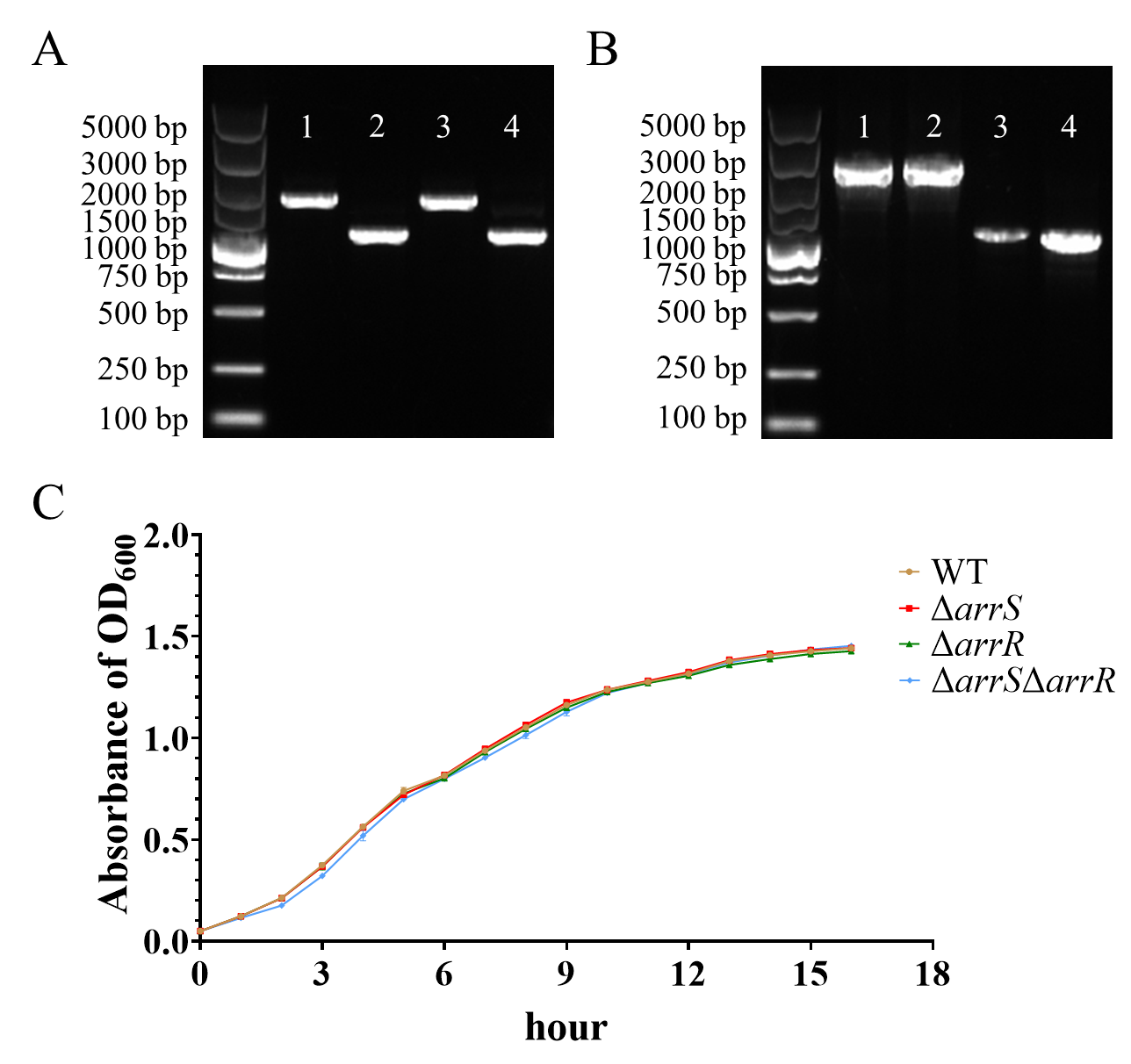


**Figure S6 Verification of *A. veronii* knockout strains and their growth curves.** *A.veronii* WT, Δ*arrS*, Δ*arrR* and Δ*arrSarrR* (numbered 1 to 4 respectively) were confirmed by PCR using primers *arrS*-F0/R0 in **(A)** and *arrR*-F0/R0 in **(B)**. And their growth curves were determined in LB using a microplate reader and presented in **(C)**. Experiments were conducted with five replicates in **(C)**. P-values were obtained from t-tests and the error bars indicate the standard deviation.


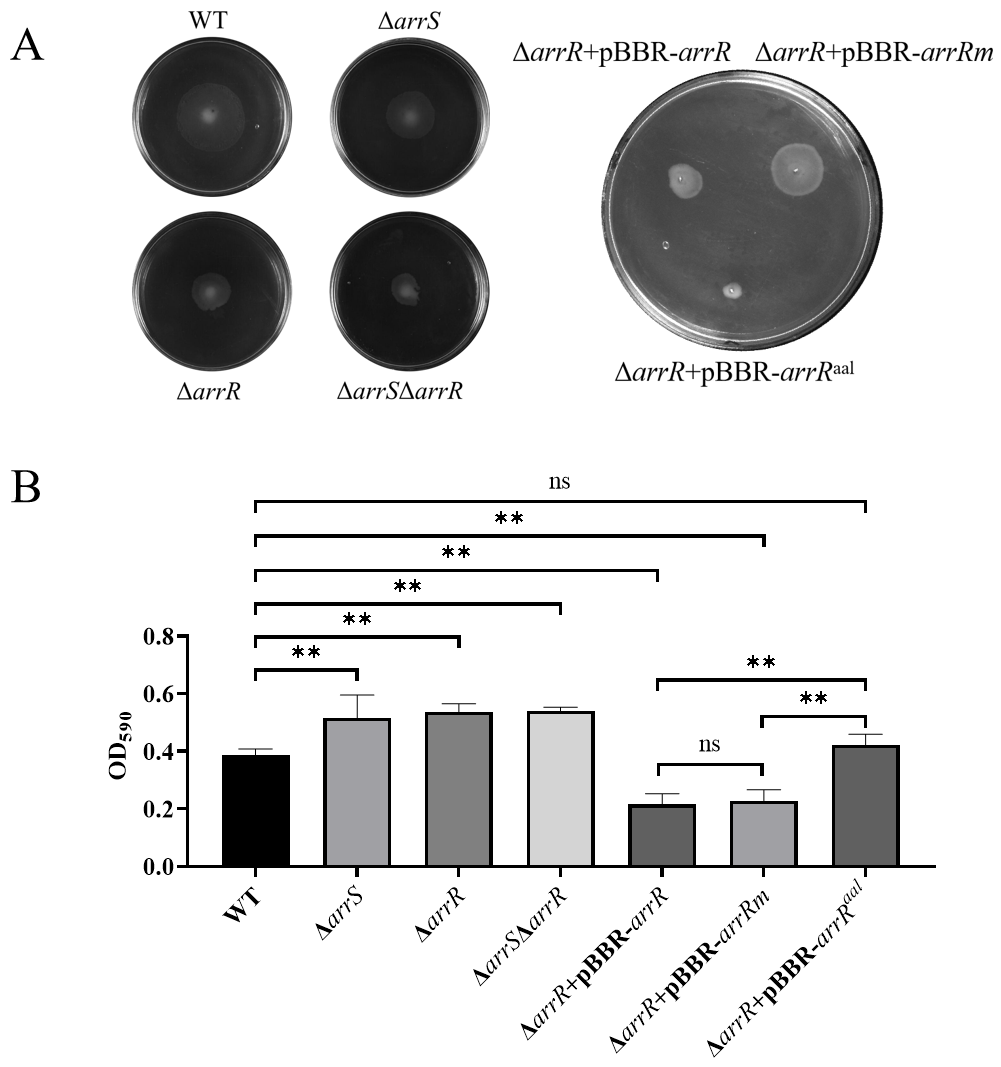


**Figure S7 Verification of intracellular c-di-GMP levels through phenotypic analysis. (A)** swimming motility. **(B)** biofilm formation.

**Figure S8 Point mutations in ARG-BOX maintain the original activity of P*_arrS_*.** Point mutations at ARG-BOX of P*_arrS_* were induced by P*_arrS_*m. And the expression of GFP was regulated by P*_arrS_* in pDH114 and P*_arrS_*m in pDH114m. Fluorescence intensity observed in *E. coli* XL1-Blue MRF' carrying pDH114 and pDH114m indicated their promoter activity. The experiment was performed with three replicates. P-values were obtained from t-tests and the error bars indicate the standard deviation.
